# Nucleolar Association and Transcriptional Inhibition through 5S rDNA in Mammals

**DOI:** 10.1371/journal.pgen.1002468

**Published:** 2012-01-19

**Authors:** Andrew M. Fedoriw, Joshua Starmer, Della Yee, Terry Magnuson

**Affiliations:** Department of Genetics, Carolina Center for Genome Sciences, Lineberger Comprehensive Cancer Center, University of North Carolina at Chapel Hill, Chapel Hill, North Carolina, United States of America; Rosalind Franklin University of Medicine and Science, United States of America

## Abstract

Changes in the spatial positioning of genes within the mammalian nucleus have been associated with transcriptional differences and thus have been hypothesized as a mode of regulation. In particular, the localization of genes to the nuclear and nucleolar peripheries is associated with transcriptional repression. However, the mechanistic basis, including the pertinent *cis-* elements, for such associations remains largely unknown. Here, we provide evidence that demonstrates a 119 bp 5S rDNA can influence nucleolar association in mammals. We found that integration of transgenes with 5S rDNA significantly increases the association of the host region with the nucleolus, and their degree of association correlates strongly with repression of a linked reporter gene. We further show that this mechanism may be functional in endogenous contexts: pseudogenes derived from 5S rDNA show biased conservation of their internal transcription factor binding sites and, in some cases, are frequently associated with the nucleolus. These results demonstrate that 5S rDNA sequence can significantly contribute to the positioning of a locus and suggest a novel, endogenous mechanism for nuclear organization in mammals.

## Author Summary

Eukaryotic genomes are compartmentalized within nuclei such that physiological events, including transcription and DNA replication, can efficiently occur. The mechanisms that regulate this organization represent an exciting, and equally enigmatic, subject of research. In mammals, the identification of elements that influence these associations has been impeded by the complex nature of the genomes. Here, we report the identification and characterization of such an element. We demonstrate that the integration of a 5S rDNA gene, a 119 base pair noncoding RNA transcribed by RNA polymerase III, into a new genomic location can significantly influence the association of the host region with the nucleolus. This positioning has drastic, inhibitory effects on the transcription of a neighboring protein coding gene transcribed by RNA polymerase II, demonstrating a functional relationship between localization and gene expression. We also provide data that suggest this may be an endogenous phenomenon, through a class of repetitive sequences derived from 5S rDNA. Together, our data not only demonstrate a structural role for 5S rDNA but also suggest that nuclear organization of mammalian genomes may be strongly influenced by repetitive sequences.

## Introduction

The organization of DNA within mammalian nuclei is considered nonrandom [1]. A number of characteristics have been proposed to influence the position of a gene or chromosomal region within the nucleus, including gene density and transcriptional activity [2]. However, the parameters that drive nuclear organization are likely complex and remain largely enigmatic. Significant proportions of mammalian genomes are comprised of noncoding, repetitive elements, many of which are derived from RNA polymerase III (pol III) transcripts. An increasing number of examples have suggested diverse roles for repetitive elements in modulating transcription of neighboring protein-coding genes transcribed by RNA polymerase II (pol II) [3], [4], [5], [6]. In yeast, binding sites for the pol III transcription factor complex, TFIIIC, play a significant role in chromatin structure and nuclear organization: tRNA genes and tRNA-like sequences function as chromatin barriers to prevent the spread of heterochromatin, while in other contexts these elements cluster together often at the nuclear and nucleolar peripheries [7], [8]. This latter phenomenon typically results in silencing of nearby pol II-transcribed genes [9].

**Figure 1:**
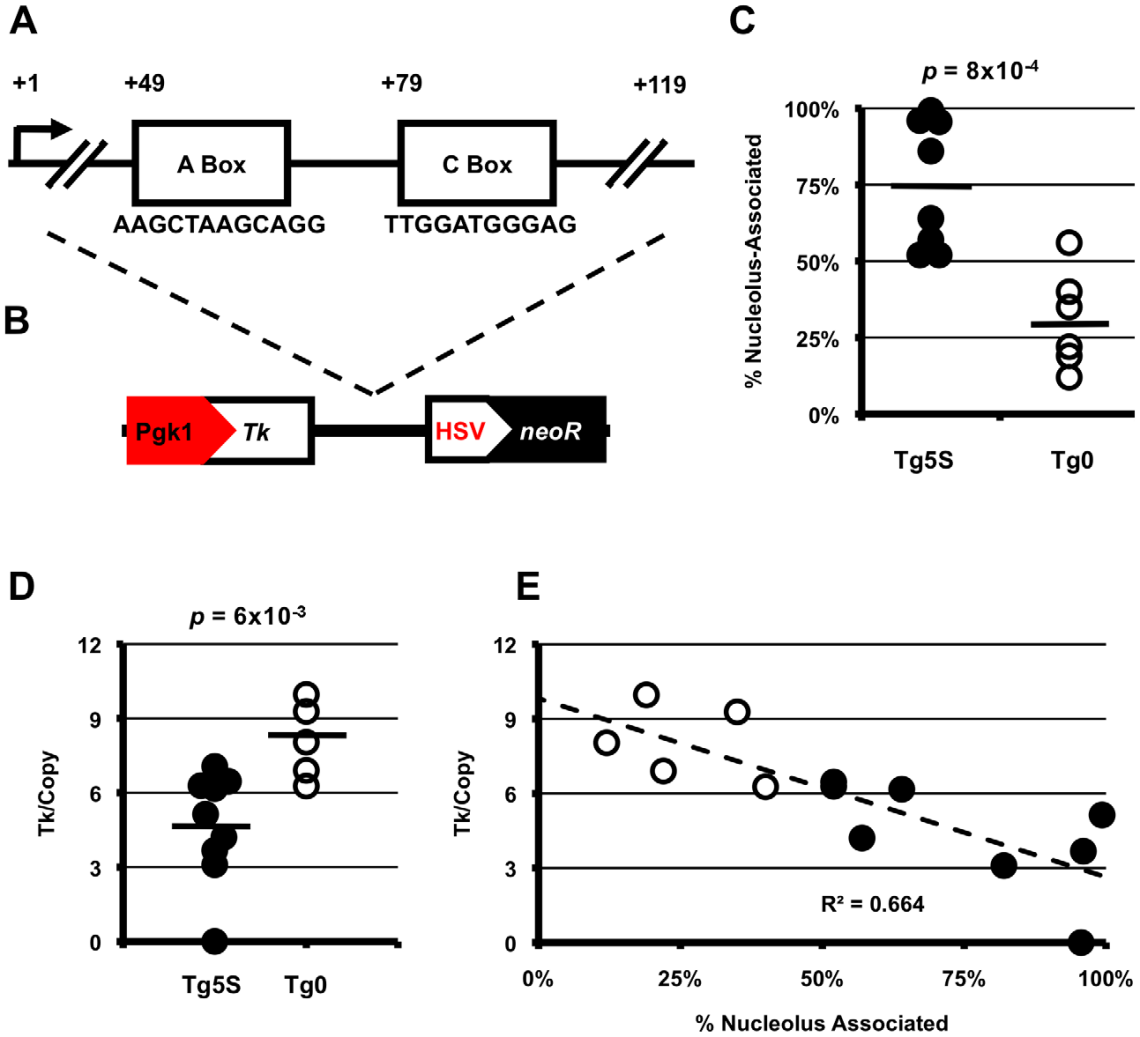
5S rDNA transgenes show preferential association with the nucleolus and decreased transcription of a reporter gene. A. Schematic of 5S rRNA gene structure showing the sequence of the highly conserved A and C boxes. B. Schematic of the transgene used in this study with position of 5S rRNA sequence relative to *NeoR* selectable marker and *Tk* reporter gene. C. Summary of nucleolar associations for transgenes with 5S rDNA (Tg5S, *n* = 9) or empty-vector transgenes (Tg0, *n* = 6). D. *Tk* mRNA levels normalized for copy number. For (C) and (D), black bars represent averages for each category. Significance determined by two-tailed t-test. E. Copy-number normalized *Tk* mRNA levels (y-axis) plotted against frequency of nucleolar association.

Moreover, just as pol II genes are thought to cluster in transcription ‘factories’ [10], active pol III also forms distinct foci in mammalian nuclei that contain a number of active pol III genes [11]. Since most pol III transcribed genes, including those of repetitive elements, carry internal promoters, they could confer intrinsic structural and regulatory properties to the surrounding genomic sequence upon insertion. Given their widespread and nonuniform distribution in mammalian genomes through repetitive elements, pol III promoters may have significant influence on chromatin structure. Furthermore, binding sites for pol III transcription factors within these elements may be under positive selection if beneficial for host genome fitness. To test these hypotheses, we focused on 5S rRNA genes (Figure 1A), which have long been known to possess unique qualities with regard to chromatin structure. We use a number of complimentary approaches to demonstrate that ectopic 5S rDNA sequence can mediate nucleolar association of a genomic region, with significant effects on local transcription. We also provide evidence that this mechanism may be active in endogenous contexts in the mouse genome: psuedogenes that are derived from 5S rDNA show preferential conservation of internal transcription factor binding sites can be bound by TFIIIC and localize to the nucleolar periphery.

## Results

A well-known nucleosome positioning sequence, 5S rDNA genes (endogenously present as multi-copy arrays in most eukaryotic genomes) have been observed to form large chromatin loops in *Xenopus* and mammalian systems [12], [13]. In agreement with observations in other eukaryotes, and recently published descriptions of chromatin associated with nucleoli in human cells [14], [15], [16], we found the mouse 5S rDNA gene array (located on the distal end of chromosome 8) associated with the nucleolar periphery in ∼40% of mouse embryonic stem (ES) cells (Figure S1A). If localization to the nucleolar periphery is an intrinsic quality of the 5S rRNA genes, then *de novo* insertion of these sequences into new genomic contexts should recapitulate this phenomenon. To study the effect of 5S rDNA sequence on sub-nuclear localization, we generated ES cell lines with stable, multicopy insertions of a reporter construct containing a single 5S rRNA gene (Tg5S) (Figure 1B). To determine whether transgenes with 5S sequence would be found at the nucleolar periphery, we then assessed localization of the stable transgenes by DNA FISH with a probe for the vector backbone relative to immunofluoresence against Nucleolin, a marker for the nucleolus [17]. In support of our hypothesis, we observed significantly more frequent localization to the nucleolar periphery of Tg5S (75%) compared with empty vector controls (Tg0, 31%, *p* = 8×10^−4^, Figure 1C, Figure S1B–S1D). Strikingly, several lines showed nearly constitutive association of the Tg5S signal with the nucleolus. This was not simply a reflection of copy number, as this pattern of localization was observed in both high- and low-copy Tg5S lines (Figure S1E, R^2^ = 0.087). Furthermore, association of Tg5S was higher than that of the 5S rDNA array (∼40%). This could be due to a dominant localization pattern imparted by Tg5S even at low copy, or additional forces acting to constrain localization of the endogenous 5S rDNA locus. We observed very little co-localization of Tg5S arrays and the 5S rDNA cluster (<1%, data not shown), demonstrating that these loci do not occupy the same compartment in the nucleoplasm. However, we found that the structural and functional integrity of the nucleolus was essential for localization through 5S rDNA. Reorganization of nucleolar components, through pharmocological inhibition of RNA polymerase I activity, resulted in a significant decrease of both Tg5S and 5S rDNA association with the nucleolus (Figure S2).

The nucleolar periphery has typically been thought of as a transcriptionally quiescent compartment, often associated with examples of constitutive [18], [19], [20] and facultative [21], [22], [23] heterochromatin. To study the effects of nucleolar association through the 5S rDNA mechanism on pol II transcription, we quantified mRNA levels of a reporter gene present on the vector: the *Thymidine kinase* (*Tk*) gene driven by the mouse *Pgk1* promoter (Figure 1B). *Tk* mRNA levels, when normalized for copy number, are significantly decreased in Tg5S lines compared with Tg0 lines (4.68±2.22 and 8.09±1.55 arbitrary units, respectively; *p* = 6×10^−3^, Figure 1D, Figure S3). Interestingly, *Tk* mRNA levels show a strong negative correlation with nucleolar association: lines with the most frequent association had the lowest normalized expression (Figure 1E, R^2^ = 0.664). This relationship suggests that perinucleolar targeting of transgenes via the 5S rDNA sequence has inhibitory effects on pol II transcription.

**Figure 2:**
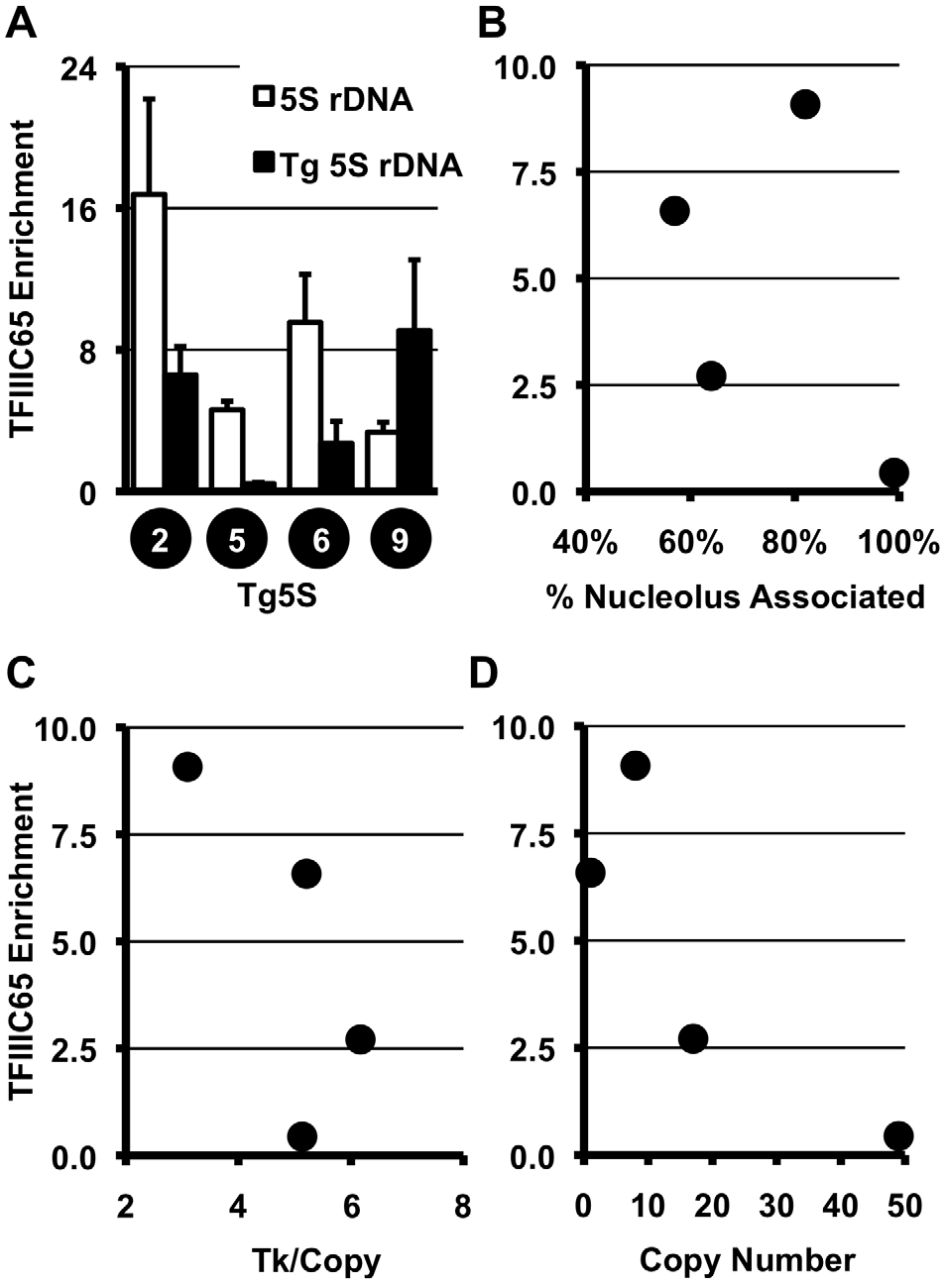
TFIIIC complex association and histone modifications at 5S rDNA transgenes. A. ChIP for the TFIIIC component, TFIIIC65, shows significant association with endogenous and transgene-5S rDNA (Tg5S rDNA). In all cases except Tg5S#5, *p*<0.05 (two tailed t-test), relative to the negative control, the *Ascl2* promoter. Relationship between TFIIIC65 enrichment and nucleolar association (B), *Tk* mRNA levels (C), and Tg5S copy number (D).

The efficiency of nucleolar localization and transcriptional repression observed by Tg5S may be related to its ability to recruit the pol III transcriptional machinery. In yeast, the regulatory capacity of tRNA and tRNA-like sequences is dependent upon the TFIIIC complex [14]. To determine whether the TFIIIC complex is associated with transgene-5S rDNA, we used chromatin immunoprecipitation (ChIP) for a subunit of TFIIIC, TFIIIC65. We observed significant levels of TFIIIC65 association with transgene-5S rDNA, relative to the negative control (the *Ascl2* promoter), in three of four Tg5S lines analyzed (Figure 2A). However, TFIIIC65 enrichment showed no clear correlation with localization (Figure 2B), *Tk* mRNA levels (Figure 2C), or copy number (Figure 2D). These data suggest that while the TFIIIC complex may participate in the localization and transcriptional attenuation we have observed for the 5S transgenes, levels of TFIIIC65 alone are not sufficient to explain these phenomena.

**Figure 3:**
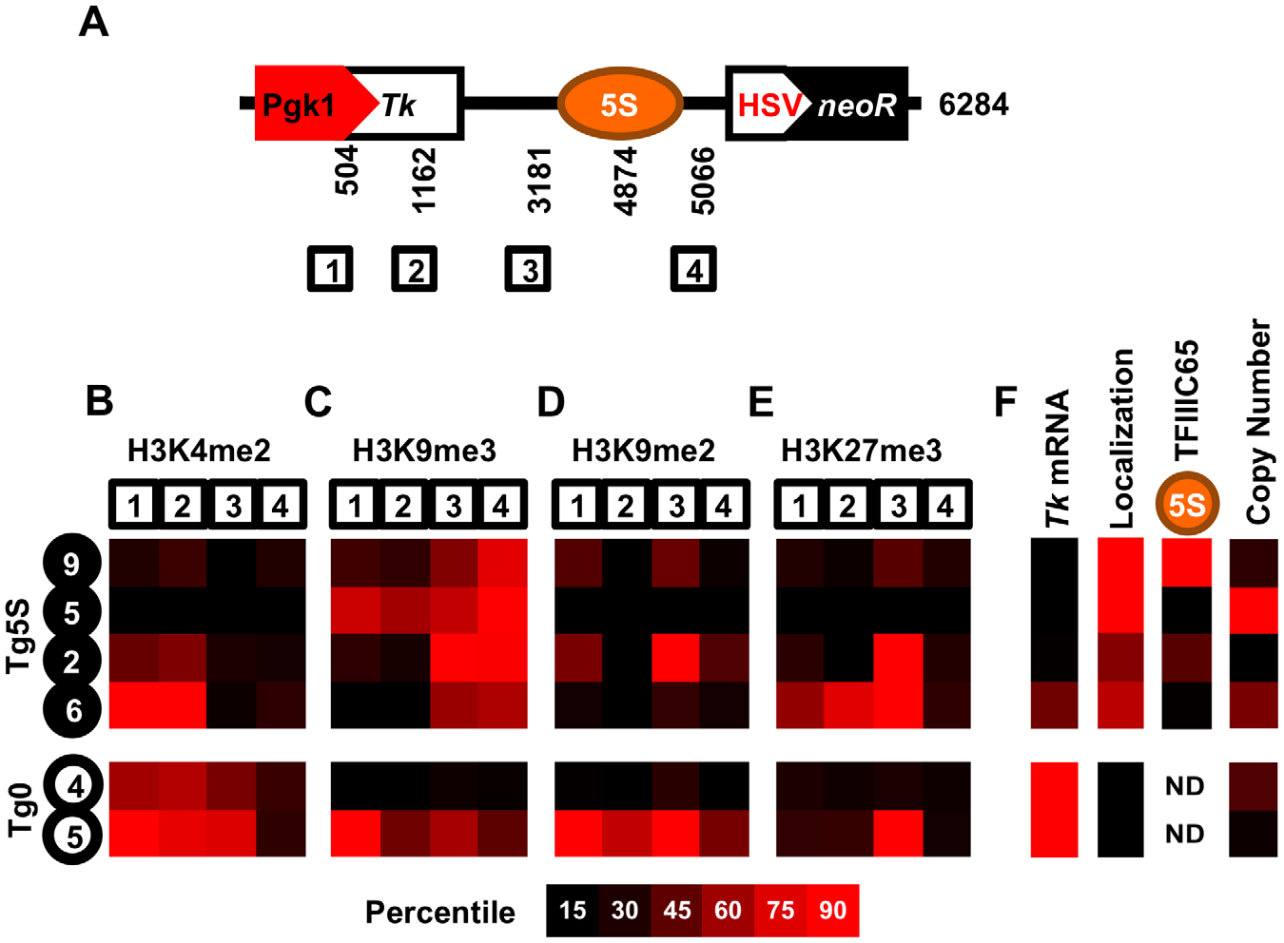
Schematic of the transgene. A. Schematic of the transgene showing the four regions analyzed for histone modifications, along with their base-pair position relative to the 5′ end of the transgene. We determined enrichment of H3K4me2 (B), H3K9me3 (C), H3K9me2 (D), and H3K27me3 (E) in four Tg5S lines (black circles) and two control Tg0 lines (white circles). Each heatmap illustrates the relative enrichment of that modification at each position in each line. For comparison, heatmaps of *Tk* mRNA levels, nucleolar localization, TFIIIC65 enrichment, and copy number for each line are shown in (F). ND, not determined.

To determine whether specific histone modifications characterize the presence of a 5S rDNA, we surveyed the distribution of several modifications at various positions within the transgenes (Figure 3A). We analyzed one mark of active chromatin (H3K4me2, Figure 3B, Figure S4A), one mark of constitutive heterochromatin (H3K9me3, Figure 3C, Figure S4B), and two marks of facultative heterochromatin (H3K9me2 and H3K27me3, Figure 3D, 3E, Figure S4C, S4D), in four Tg5S and two Tg0 cell lines. As expected, cell lines with higher expression of *Tk* (Figure 3F) had increased levels of H3K4me2 at the *Tk* gene. Intriguingly, all Tg5S lines were characterized by high levels of H3K9me3 near the 5S rDNA, rather than the *Tk* gene body or promoter. Both patterns were evident irrespective of TFIIIC65 enrichment to the transgene-5S rDNA (Figure 3F). These observations suggest an association between the 5S rDNA sequence and the H3K9me3 modification.

**Figure 4:**
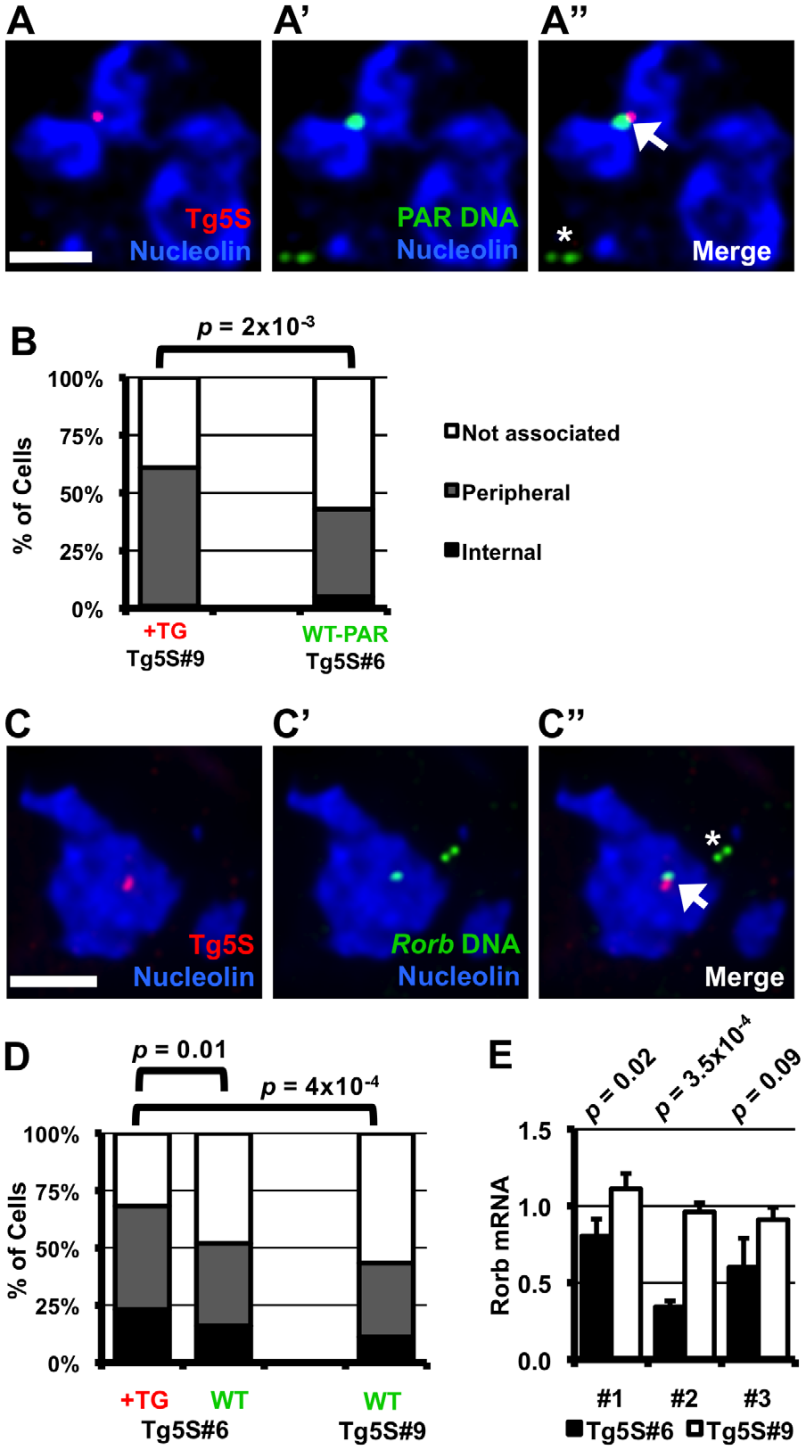
Nucleolar association of a genomic region increases upon integration of 5S rDNA transgenes. A–A″. DNA FISH in Tg5S line #9 (Tg5S#9) for transgene (red, A), and PAR (green, A′), along with IF for Nucleolin (blue). In A″, the arrowhead indicates the allele with transgene integration, the asterisk indicates the wild-type allele. B. Nucleolar association of PARs with Tg5S integration in Tg5S#9 (*n* = 39), and a wild-type X chromosome PAR in line Tg5S#6 (*n* = 61). C. DNA FISH showing Tg5S integration in the *Rorb* locus on chromosome 19 in Tg5S#6 (C–C″). D. Localization of *Rorb* alleles with (+TG, *n* = 82) and without (WT, *n* = 75) Tg5S integrations in Tg5S#6, and the cumulative localization of both alleles in a control line (Tg5S#9, *n* = 62). For analysis, deconvolved Z-stacks were rendered as 3-dimensional models; for illustration, each image is a Z-stack projection (see Methods Summary). Statistical significance was determined by chi-squared test. Scale bars are 2 µm. E. *Rorb* expression in differentiated Tg5S#6 and Tg5S#9. Each pair represents a biological replicate of retinoic-acid induced differentiation. Statistical significance was determined by t-test; *p*-values are shown above each replicate.

The frequent nucleolar association of 5S rDNA-containing transgenes suggests the capacity to direct localization of a genomic region to the nucleolar periphery. However, this observation may also reflect preferential integration of Tg5S into a chromosomal region neighboring the nucleolus in the parental cells, rather than a change in localization. To discriminate between these possibilities, we identified the insertion site for several Tg5S ES lines. We mapped the transgene insertion in Tg5S#9 to the pseudoautosomal region (PAR) of the X chromosome [24] (Figure S5A). Since these ES cells are XY, we used the X-chromosome PAR of a line without a transgene insertion in this region as a control (Tg5S#6) to assess localization changes relative to a homolgous, wild-type chromosome. The PAR with the transgene insertion was more frequently associated with the nucleolus (61%) than a wild-type PAR (43%, *p* = 2×10^−3^, Figure 4A, 4B). Although nucleolar association of the wt PAR was similar to that of the 5S rDNA locus (39%), this frequency increased significantly upon Tg5S insertion.

Tg5S line #6 (Tg5S#6), contains an integration in the first intron of the silent *RAR-related orphan receptor beta* (*Rorb*) gene (Figure S5B). The allele containing the transgene array was discernable by DNA FISH and always overlapped with the genomic probe (Figure 4C). Nucleolar association was measured for both the wild type allele (wt allele) and the allele containing the Tg5S insertion (tg allele). As a control, we measured localization of the *Rorb* alleles in Tg5S#9, which does not have an insertion in this region. We detected significantly more DNA FISH signals for the tg allele associated with or internal to the nucleolus (68%) than for the wt allele (52%) in Tg5S#6 (Figure 4D, *p* = 0.01), or either allele in the control cell line (43%, *p* = 4×10^−4^). The localization frequency of the wt allele in the Tg5S#6 was not significantly different from the alleles in the control line (*p* = 0.5). Interestingly, wt *Rorb* alleles were associated with the nucleolus significantly more frequently than the 5S rDNA locus (chi-squared test, *p* = 5×10^−9^). Together, our observations from two independent insertion events, in two very different genomic contexts, demonstrate that ectopic 5S rDNA can influence the position of a locus.

Since localization by a Tg5S was associated with decreased transcriptional output of the *Tk* reporter gene, we hypothesized that the transgene insertion into the *Rorb* locus may similarly affect transcription of this gene. *Rorb* is not expressed in undifferentiated ES cells, therefore we differentiated the line with the Tg5S insertion in the *Rorb* gene (Tg5S#6) along with Tg5S#9, where the transgene insertion is not at the *Rorb* locus. Although activation of *Rorb* was variable between biological replicates, in each case *Rorb* expression was significantly reduced in Tg5S#6 (Figure 4E). Intriguingly, average *Rorb* expression in Tg5S#6 was 60% of that in Tg5S#9, suggesting that the presence of Tg5S at the *Rorb* locus has detrimental effects on its transcriptional activation.

**Figure 5:**
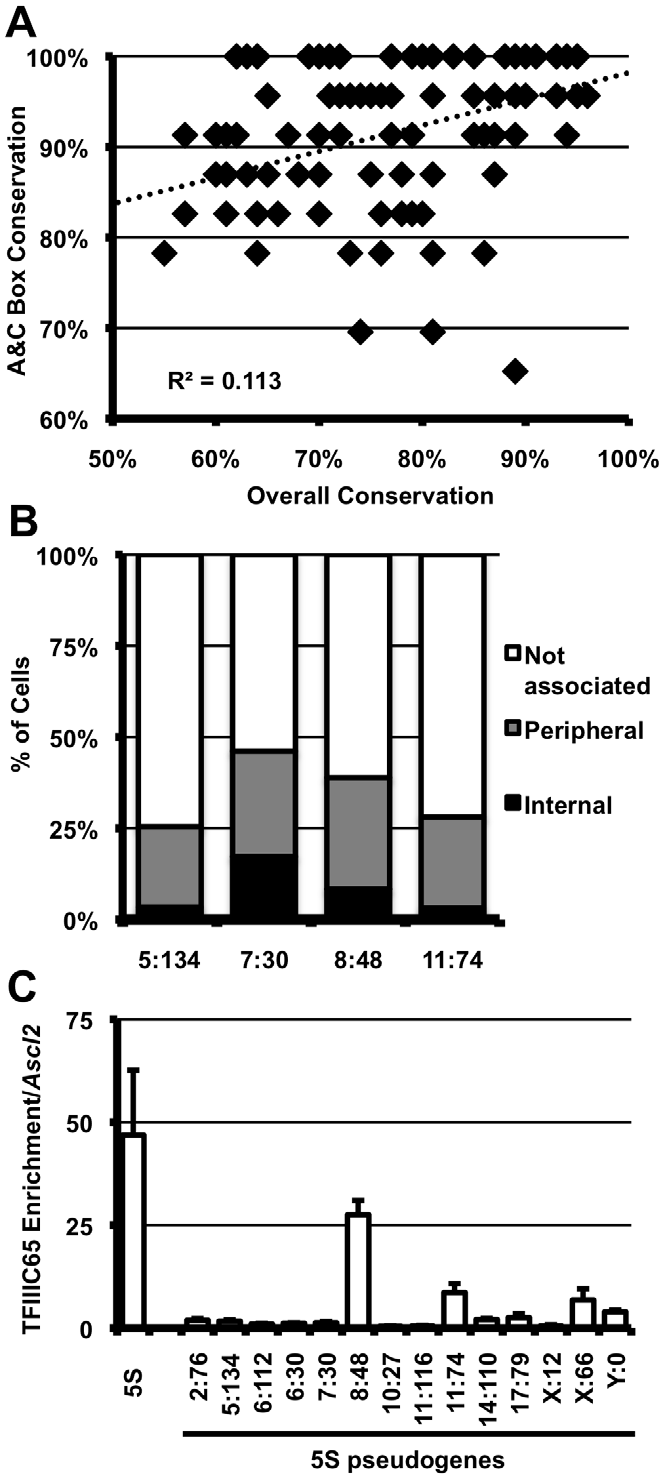
5S rDNA pseudogenes with conserved internal binding sites are associated with the nucleolus and bound by TFIIIC. A. Conservation within the A and C boxes (y-axis) relative to overall conservation of 5S pseudogenes (x-axis) in the mouse genome. Each diamond represents a single peudogene. B. Nucleolar association of pseudogenes; pseudogenes are labeled by their location in the genome as chromosome∶megabase (5∶134, *n* = 59; 7∶30, *n* = 51; 8∶48, *n* = 35; 11∶74, *n* = 32). Single focal sections were analyzed, and scored if at least one signal was internal or peripheral to the nucleolus. C. TFIIIC65 enrichment at 5S pseudogenes in E14 ES cells. Data are represented as fold enrichment of 5S pseudogene relative to enrichment of the negative control, the *Ascl2* promoter, in the TFIIIC65 ChIP.

The mouse genome contains >110 5S rDNA genes annotated outside the array on chromosome 8 (NCBI m37 mouse assembly, Table S1, Figure S6A). However, these elements show low overall sequence conservation and no predicted structural similarity to *bona fide* 5S rDNA, and are therefore unlikely to be functional components of the large ribosomal subunit (Figure S6B, S6C). Despite acquiring numerous mutations, a high proportion of these 5S pseudogenes retain perfect, or near-perfect, internal transcription factor binding sites (Figure 1A). This conservation correlates poorly with overall similarity of the 5S pseudogenes to the 5S rDNA consensus (R^2^ = 0.113, Figure 5A), suggesting this is not simply due to recent insertion events, but rather indicative of differential selective pressure within the psuedogene. We found a subset 5S pseudogene loci associated with the nucleolus in E14 ES cells at a frequency comprable to that of the 5S rDNA locus (Figure 5B, Figure S7), further supporting a positional effect for this sequence. TFIIIC association with pseudogenes was not well correlated with localization: by ChIP, we observed high levels of TFIIIC65 enrichment at only one of two pseudogene loci frequently associated with the nucleolus (Figure 5C). Therefore, if nucleolar association of these regions is mediated through 5S pseudogenes, then it may not require stable association of the TFIIIC complex, or perhaps involve altogether different mechanisms. Irrespective of the putative *trans*-factor, frequent nucleolar association of 5S pseudogenes further support a previously uncharacterized role for for these sequences as organizational *cis*-elements in the mammalian genome.

## Discussion

**Figure 6:**
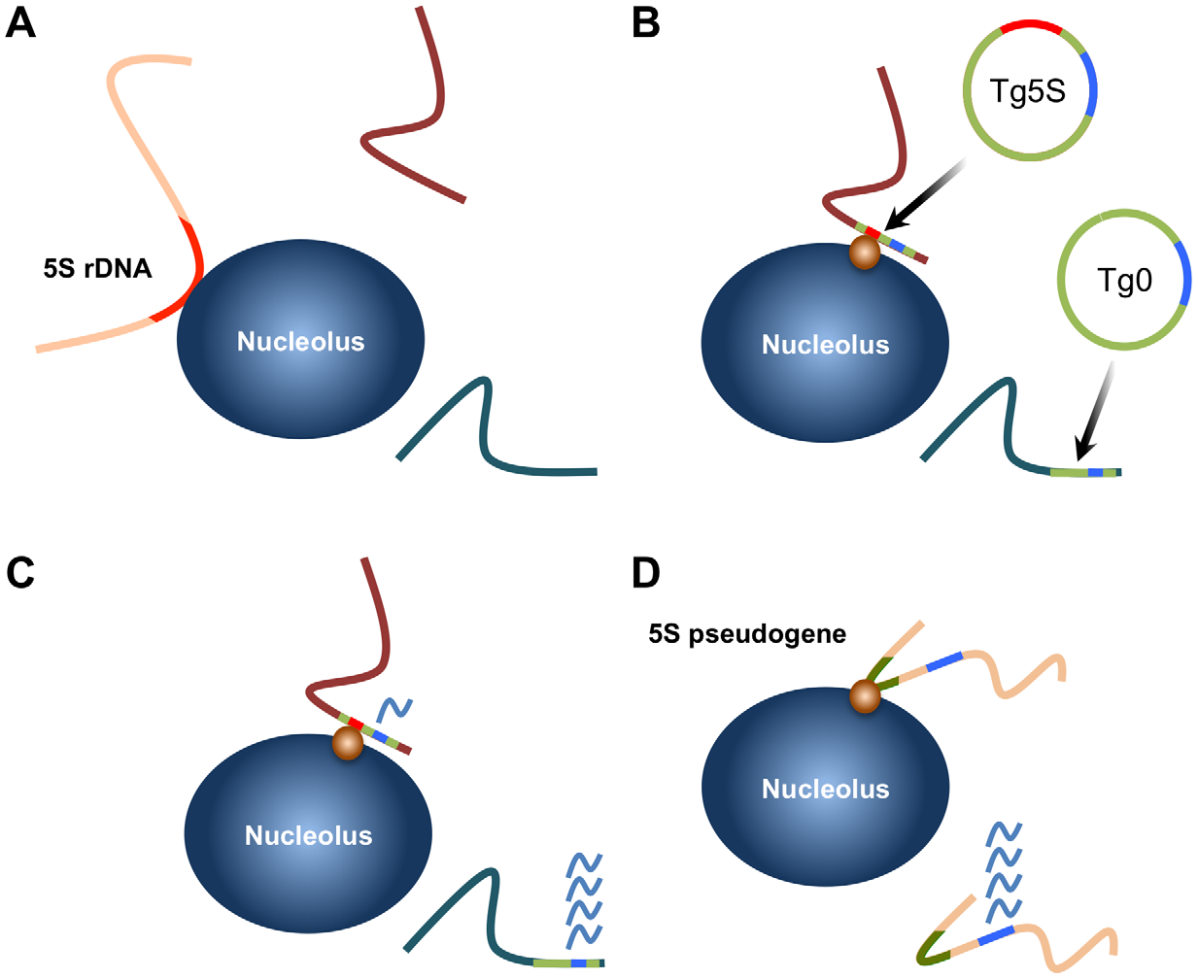
Summary of localization by 5S rDNA and transgenes and model for gene regulation by 5S pseudogenes. A. The 5S rRNA gene array (located on chromosome 8) is associated with the nucleolus in ∼40% of ES cells. B. 5S rDNA integrated at ectopic positions as transgenes are frequently associated with the nucleolar periphery (Tg5S; 5S rDNA is represented by the red line). Furthermore, integration of Tg5S increases nucleolar association of the host locus (purple line). Control vectors (Tg0) do not show this preferential association. This positioning likely depends on *trans*-factors (orange circle), potentially RNA pol III transcription factors. C. Transcription of a pol II-driven reporter gene (blue line) is reduced from Tg5S lines relative to vectors lacking the 5S rDNA sequence. The repressive effect observed in Tg5S lines strongly correlates with nucleolar association frequency. D. We observed that a subset of 5S pseudogenes (olive lines) are also associated with the nucleolus. Based on our results, we hypothesize that nucleolar association of a pseudogene would reflect a repressive effect on transcription of nearby protein coding genes (blue lines), through the same mechanism as Tg5S.

The relationship between the organization of chromatin within the nucleus and the regulation of individual genes has become an intensely studied subject. However, the complex nature of mammalian genomes has largely confounded efforts to understand the nature of this relationship. Several reports have catalogued the DNA and chromatin associated with the nuclear lamina and nucleolar periphery [15], [16], [25]. These findings have identified common characteristics of each domain, yet the basis for their presence at these compartments has remained less clear. Other studies have utilized fusion proteins to artificially tether *lacO* arrays to the nuclear lamina and other nuclear bodies [26], [27], [28]. Conversely, we have identified an endogenous sequence element, utilizing native nuclear machinery, that is capable of influencing subnuclear position. While transgenes with binding sites for the vertebrate insulator protein CTCF [29] have been shown to associate with nucleoli in a CTCF-dependent manner [30], it is not known how frequently endogenous CTCF sites recapitulate this phenomenon. Our data demonstrate that 5S rDNA sequence can confer a positional bias in localization, and correlates with an attentuation of nearby pol II transcription (summarized in Figure 6). Importantly, the localization of 5S rDNA pseudogenes to the nucleolar periphery suggest this event is not limited to ectopic transgene integrations. Biased conservation of transcription factor binding sites within 5S pseudogenes implies a functional role in their endogenous contexts. We propose that the internal transcription factor sites of 5S rDNA represents a novel, *cis-* acting influence of nuclear position in mammals. This hypothesis is supported by the observed enrichment of 5S rDNA sequences in nucleolar-associated chromatin of human cells [15], [16].

Recently, genome-wide maps of pol III and associated transcription factor binding in human cells have suggested structural roles reminiscent of what has been observed in yeast. These studies identified a number of “extra-TFIIIC” (ETC) loci, TFIIIC-bound regions not associated with a pol III complex or transcription unit [31], [32]. However, unlike the ETC loci of yeast, which are associated with silencing of nearby pol II-driven promoters, human ETC loci are correlated with active pol II genes. In contrast, we observed high levels of the repressive H3K9me3 modification surrounding the 5S rDNA sequence. Thus the functional properties of ETC loci appear to be distinct from the repressive effect on pol II transcription that we have observed for 5S rDNA. Importantly, this demonstrates that presence of the TFIIIC complex alone is not sufficient to explain the effect on neighboring pol II transcription, suggesting additional or alternative factors. For example, TFIIIC recruitment to 5S rDNA first requires the binding of the TFIIIA, which specifically recognizes the A and C boxes. Alternatively, the strong nucleosome positioning properties of 5S rDNA may play a role in its localization and repressive effects on neighboring pol II transcription.

Collectively, these observations suggest broad and diverse roles for pol III genes and derived sequences in the organization of chromatin within the mammalian nucleus. Because of their number, pol III promoters may exert a stronger influence on structural organization than pol II-directed gene activity. As pol III activity is coupled with differentiation and cellular metabolism, association of pol III and transcription factors with elements such as the 5S pseudgoenes we have described, may provide the basis for global organizational and structural changes within the nucleus in response to external stimuli [33].

## Materials and Methods

### Cell Culture

E14 ES cells were cultured under standard conditions. To generate stable lines, ES cells were transfected with 1 µg of linearized plasmid using lipofectamine (Invitrogen) and selected in the presence of G418 for 14 days. We verified stable neomycin resistance for most lines by culturing with G418 and noted no increased levels of cell death. To induce differentiation, 2×10^5^ ES cells were plated on 60 mm^2^ dishes without LIF and in the presence of 0.1 µM retinoic acid (Sigma) then cultured for 8 days, with passaging to maintain low cell density. For immunofluoresence and DNA FISH, cells were plated at low density and grown on coverslips 18–24 hours. Coverslips were permeabilized with cytoskeletal (CSK) buffer (100 mM NaCl, 300 mM sucrose, 3 mM MgCl_2_, and 10 mM PIPES pH 6.8), then fixed in 4% paraformaldehyde (PFA, Electron Microscopy Sciences) for 10 minutes at room temperature, washed twice for 5 minutes in 1× PBS (Cellgro), then stored in 75% ethanol at 4°C. Coverslips were re-hydrated with several washes of 1× PBS prior to DNA FISH experiments.

### Plasmid Construction

To generate Tg5S, the 5S rDNA sequence (a gift of B.Solner-Webb, Johns-Hopkins University) was cloned into a vector that contains the neomycin resistance gene under the control of the HSV promoter, and the *Thymidine kinase* gene under the control of the mouse *Pgk1* promoter (a gift of D.Ciavatta, University of North Carolina). Tg0 was the vector without the 5S rDNA insert.

### RNA Isolation and RT–PCR

RNA was isolated from cultured cells with Trizol reagent (Invitrogen), DNAsed (RQ1 DNAse, Promega) and 500 ng of total RNA was used for each reaction. Samples were reverse transcribed using random-hexamer primers, with Superscript II Reverse Transcriptase (Invitrogen). Primers are listed in Table S2. *Tk* mRNA levels were first normalized to *Gapdh* levels. Real-time quantitative PCR was carried out on 25 ng of cDNA, in triplicate for each gene, on an ABI 3700 (Applied Biosystems), using the Fast SYBR Green Master Mix (Applied Biosystems). Data was analyzed in Microsoft Excel (Microsoft), and is shown as the log2-transformation of RNA levels relative to copy number. Statistical significance was determined by two-tailed t-test.

### Integration Site Identification

Transgene integration sites were determined using one of two approaches. The Tg5S#6 insertion site was identified using the TAIL PCR protocol, with degenerate primers as described [34]. The insertion site for Tg5S#9 was identified using inverse-PCR. Briefly, 1 µg of DNA was digested with XbaI, then ligated overnight with T4 ligase (NEB) at 2 ng/µl. DNA was concentrated by ethanol precipitation, and 50 ng of the ligation was used in a nested PCR reaction. PCR products were purified from an agarose gel using a QIAGEN gel extraction kit (QIAGEN) and quantified on a QUBIT flourometer (QIAGEN). PCR products were directly sequenced and analyzed by BLAST searches to the reference assembly of the mouse genome. Each insertion was confirmed by PCR. Primers are listed in Table S2. sequence. Each vector was linearized with XhoI (NEB) prior to lipofection.

### Immunofluoresence (IF) and DNA FISH

Coverslips were rehydrated in 1× PBS, before blocking in 10 mg/ml IgG-free BSA (Jackson Immunochemical) and 0.2% Tween-20 (Fisher) for 20–30 minutes at room temperature. Rabbit anti-Nucleolin (Bethyl Laboritories, A300-711A) was added at 1∶400 dilution into blocking buffer and incubated overnight at 4°C. Coverslips were then washed with 1× PBS, and incubated with biotinylated goat-anti-rabbit antibody, diluted in blocking buffer at 1∶400, for 2–3 hours at room temperature. Following washes with 1× PBS, cells were post-fixed with 2% PFA for 3 minutes at room temperature, washed extensively with 1× PBS, then treated with 0.01 mg/ml pepsin (Sigma) diluted in pre-warmed 0.01 N HCl for 5 minutes, and then washed extensively with 1× PBS. Following a dehydration series in ethanol, DNA was denatured in 70% formamide (Ameresco) and 2× SSC (Cellgro) for 10–20 minutes at 85°C. After several washes with cold 2× SSC, cells were incubated with prehybridized DNA FISH probes (see below) overnight at 37°C. Coverslips were washed twice with 50% formamide and 2× SSC, twice with 2× SSC (one wash had 100 ng/ml DAPI added), once with 1× SSC. To detect biotinylated secondary antibodies, coverslips were then washed once with 4× SSC for 5 minutes, incubated for 20–30 minutes in Streptavidin-647 (Invitrogen) in 2 mg/ml BSA and 4× SSC, followed by 5 minute washes of 4× SSC, 4× SSC with 0.5% Tween-20 (Fisher), and 4× SSC. All washes and incubations for biotin detection were carried out at 37°C.

### FISH probes

In addition to the vector backbone, the following BAC and fosmid probes were used in this study: BACs: 5∶134 (RP24-193L24), 7∶30 (RP23-151J21), 8∶126 (RP24-372G15), 10∶27 (RP24-213F23), 11∶74 (RP23174M12), *Mid1* (RP24-229F18); and fosmids: 6∶112 (G135P69622C7), 8∶48 (G135P60371F8, G135P60172E7), 19∶19 (G135P64778C12), PAR (G135P601180H2) (all clones were acquired from CHORI BPRC). BACs and fosmids were isolated by a standard alkaline lysis protocol. Approximately 25 ng of DNA was labeled with BioPrime DNA labeling kit (Invitrogen), using FITC-conjugated dUTP (Roche), Cy3- or Cy5-conjugated dCTP (GE Healthcare), and stored in 70% ethanol at −20°C. To prepare FISH probes for hybridization, probes were precipitated with mouse Cot-1 DNA (Invitrogen), yeast tRNA (Invitrogen), and Salmon Sperm DNA (Invitrogen). After washes with 75% and 100% ethanol, probes were air-dried and denatured for 10 minutes in 50–100 µl of 100% formamide at 85°C. An equal volume of 2× hybridization buffer (25% dextran sulfate/4× SSC) was then added, and probes were pre-hybridized for 60 to 90 minutes at 37°C. Probes were stored at −20°C until use.

### Microscopy and Image Analysis

IF-DNA FISH was carried out as described in Methods. For transgene-nucleolus association, cells were visualized on Leica DMLB fluorescent microscope (Leica), captured on a Retiga 2000R Fast camera (Qimaging), using QCapture software (Qimaging), and merged with Adobe Photoshop (Adobe). DNA FISH signals were considered ‘nucleolar associated’ if the FISH signals were in contact with, or within, the Nucleolin signal. For determining nucleolar association of 5S rDNA, pseudogenes, and genomic loci with transgene insertions, Z-stacks of each channel were taken on a Ziess AxioImager M2 microscope, deconvolved using the Axiovision software package (Zeiss), then rendered in 3-dimensions using the ZEN Light Edition 2009 software (Zeiss). Signals were considered ‘internal’, if the center of the FISH signal was internal to the Nucleolin signal; ‘peripheral’ if the pixels of the FISH and Nucleolin signals were overlapping, but the center of the FISH signal was outside; and ‘not associated’ if there was visible distance between the DNA FISH signal and the outside of the Nucleolin-labeled nucleolus. Statistical significance was determined by chi-squared.

### Copy Number Determination

Copy number was determined by quantitative PCR to determine the number of *Neo* gene copies relative to an endogenous locus (the *Ascl2* promoter), then normalized to a genomic DNA sample containing 1 copy of *Neo* for each diploid genome. Primers are listed in Table S2.

### Chromatin Immunoprecipitation (ChIP)

ES cells were trypsinized, counted, resuspended at 10^7^ cells/ml and fixed with 1% formaldehyde. After quenching with 0.125 M glycine, cells were pelleted, washed once with cold 1×PBS, pelleted again and used for ChIP or frozen at −80°C. Protease inhibitors (Sigma) and PMSF (Sigma) were added to all steps until washing steps. For GTF3C5 and pseudogenes, ChIP was performed as described [35]. To measure histone modificiations or GTF3C5 association with transgenes, cell pellets were resuspended in solution L1 (50 mM HEPES-KOH, pH 7.5, 140 mM NaCl, 1 mM EDTA, 10% glycerol, 0.5% NP-40, 0.25% Triton X-100) at 10^7^ cells/ml, mixed at 15 minutes and gently pelleted at 4°C. Cell pellet was resuspended in solution L2 (10 mM Tris-HCl, pH 8.0, 200 mM NaCl, 1 mM EDTA, 0.5 mM EGTA) at 10^7^ cells/ml, mixed at 15 minutes and gently pelleted at 4°C. Cells were lysed in solution L3 (10 mM Tris-HCl, pH 8.0, 100 mM NaCl, 1 mM EDTA, 0.5 mM EGTA, 0.1% Na-Deoxycholate, 0.5% N-lauroylsarcosine) for 10 minutes at 4°C. Chromatin was sheared by sonication to generate fragments 2–600 bp. Before immunoprecipitation, 1/10^th^ of each sample was removed as ‘input’. 5 µg of Antibody (rabbit anti-GTF3C5, A301-242A, Bethyl Laboratories; rabbit anti-H3K4me2, 07-030 Millipore; mouse anti-H3K9me2, ab1220, Abcam; rabbit anti-H3K9me3, ab8898, Abcam; or mouse anti-H3L27me3, ab6002, Abcam) or normal rabbit sera (Abcam) was conjugated to protein A/G beads in 0.5%BSA/1×PBS overnight at 4°C on a nutating platform. Chromatin was incubated with bead-conjugated primary antibody overnight at 4°C with gentle mixing. For GTF3C5 ChIP, beads were then washed for 5 minutes at 4°C with gentle mixing, using the following solutions: Low Salt Buffer (0.1% SDS, 1% Triton X-100, 2 mM EDTA, 20 mM Tris, 150 mM NaCl), twice; High Salt Buffer(0.1% SDS, 1% Triton X-100, 2 mM EDTA, 20 mM Tris, 500 mM NaCl), once; LiCl buffer (1 mM EDTA, 10 mM Tris, 250 mM LiCl, 1% NP-40, 1% Na-Deoxycholate), twice; and TE (10 mM Tris, 1 mM EDTA), twice. For histone modifications, beads were washed 4 times with RIPA buffer, and once with TE containing 50 mM NaCl. Chromatin was eluted from beads with 2, 15-minute washes at 65°C using freshly prepared Elution Buffer (1% SDS/0.1 M NaHCO_3_). To isolate DNA, 5 M NaCl was added to pooled eluates or input chromatin to a final concentration of 0.2 M, and incubated for at least 4 hours at 65°C, then treated with 30 µg of Proteinase K (Roche) for 2 hours at 55°C. After addition of 10 µg linear acrylamide as a carrier (Ambion), DNA was extracted with 25∶24∶1 phenol∶choloform∶isoamyl alcohol (Sigma), precipitated with 100% ethanol, and resuspended in nuclease-free ddH_2_0 (Promega). For psuedogenes, three replicates of quantitative PCR were carried out on an ABI 3700 (Applied Biosystems), using the Fast SYBR Green Master Mix (Applied Biosystems). For transgene and 5S rDNA enrichment, 2–5 replicates were performed on Bio-Rad CDX96 instrument, a using SsoFast EvaGreen Supermix (Bio-Rad). PCR primers are listed in Table S2. Data are displayed as enrichment of amplicon relative to a negative control region in each ChIP. Data was analyzed in Microsoft Excel (Microsoft); statistical significance was determined by two-tailed t-test.

## Supporting Information

Figure S1 A. Combined Immunofluoresence (IF)-DNA FISH images showing localization of the 5S rRNA gene arrays in mouse ES cells. At least one allele was associated with the nucleolar periphery in ∼40% of nuclei. Examples of Tg5S nucleolar association (B) or no association (C). D. Nucleolar association of each individual Tg5S and Tg0 ES cell line. (n) is indicated below each line number. E. Relationship between association frequency (Y-axis) and copy number (X-axis). Scale bar is 2 µm.
(TIF)

Figure S2 Pharmacological inhibition of RNA polymerase I (pol I) activity results in nucleolar reorganization and a decrease in 5S rDNA and Tg5S nucleolar association. ES lines were treated with a low dose of Actinomycin D (ActD; 20 ng/ml) for 2 hours prior to fixation to inhibit Pol I elongation. Note that ActD treatment results in redistribution of the pol I transcription factor UBF1(green) from intranucleolar foci (A) into focal concentrations at the nucleolar periphery (B, white arrow), and restructuring of nucleoli into a more spherical morphology. We measured the size of ActD-treated nucleoli to be 51% smaller than untreated nucleoli. C. Localization of 5S rDNA (n = 112) and Tg5S after ActD treatment (Tg5S#5, n = 30; Tg5S#6, n = 48; Tg5S#9, n = 50). To normalize for changes in nucleolar size, we calculated the ‘expected’ localization as the frequency of association in untreated cells by the relative nucleolar size in ActD treated cells. Statistical significance was determined by comparing the expected frequency to the observed frequency by chi-squared; N.S., not significant.
(TIF)

Figure S3 Normalized *Tk* shown for each individual line. Each technical replicate is shown as a different symbol.
(TIF)

Figure S4 Quantification of histone modification enrichment over transgenes for (A) H3K4me2, (B) H3K9me3, (C) H3K9me2, and (D) H3K27me3. Values are represented as fold-enrichment relative with a negative control region lacking that modification. Also shown is a schematic of the transgene with positions of regions assayed. 2–3 replicates of each reaction were performed for each point.
(TIF)

Figure S5 A. PCR assays to genotype PAR insertion in Tg5S#9; Tg5S#2 was used as a negative control. B. PCR assay to genotype insertion of the transgene into *Rorb* allele in Tg5S#6; Tg5S#4 is shown as a negative control.
(TIF)

Figure S6 A. Distribution of 5S rDNA as annotated by in Ensembl (NCBIM37) (red dots). Perfect A and C boxes are shown as blue dots; note that a number of perfect A/C boxes are found outside of annotated 5S rDNA. The 5S rDNA array is located near the telomere of chromosome 8 (bold). Since the structure of 5S rRNA is highly conserved, we hypothesized that if the single genes were truly 5S rRNA, then they should form the expected structure. Using a folding algorithm (mfold, http://mfold.rna.albany.edu [36]), we predicted structure for all single 5S rDNA genes, and found that none had a structure resembling 5S rRNA, or thermodynamic stability (−Δ*G*), suggesting these elements are likely to be pseudogenes. Predicted 5S rRNA structure is shown in (B), while the structure of the most thermostable of 5S pseudogenes in (C).
(TIF)

Figure S7 Nucleolar association of 5S rDNA (*n* = 83) and pseudogenes: 6∶112 (*n* = 49), 8∶48 (*n* = 61), and 10∶27 (*n* = 21). Pseudogenes are labeled by their location in the genome as chromosome∶megabase. For analysis, deconvolved Z-stacks were rendered as 3-dimensional models(see Methods). Frequency for localization of 8∶48 was comprable to analysis of single focal planes (see Figure 5B; 33% peripheral, 6% internal).
(TIF)

Table S1 Mouse 5S Pseudogenes. Positional information of 5S rDNA psuedogenes in the mouse genome (based on the NCBI m37 mouse assembly). Also included are the percent identity of the A and C boxes, as well as the entire sequence, to the 5S rDNA consensus.
(DOC)

Table S2 PCR primers used in this study.
(DOC)
