# Supplementary figures and images for: Nucleolar Association and Transcriptional Inhibition through 5S rDNA in Mammals
Source: PLoS Genet. 2012 Jan 19;8(1):e1002468. doi: 10.1371/journal.pgen.1002468 (PMC3261910; doi:10.1371/journal.pgen.1002468)

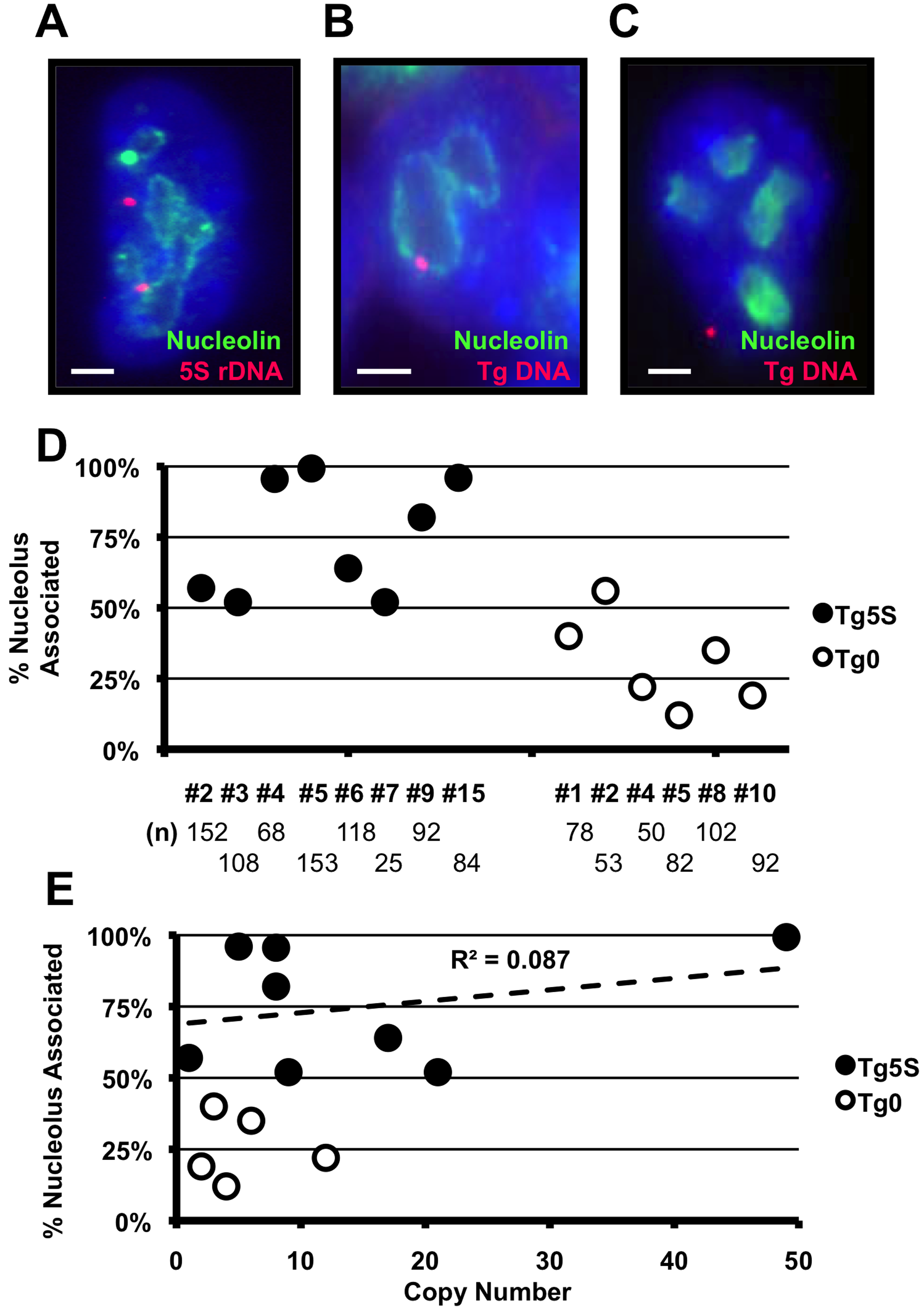

Supplement: Figure S1 — A. Combined Immunofluoresence (IF)-DNA FISH images showing localization of the 5S rRNA gene arrays in mouse ES cells. At least one allele was associated with the nucleolar periphery in ∼40% of nuclei. Examples of Tg5S nucleolar association (B) or no association (C). D. Nucleolar association of each individual Tg5S and Tg0 ES cell line. (n) is indicated below each line number. E. Relationship between association frequency (Y-axis) and copy number (X-axis). Scale bar is 2 µm. (TIF) [file pgen.1002468.s001.tif]

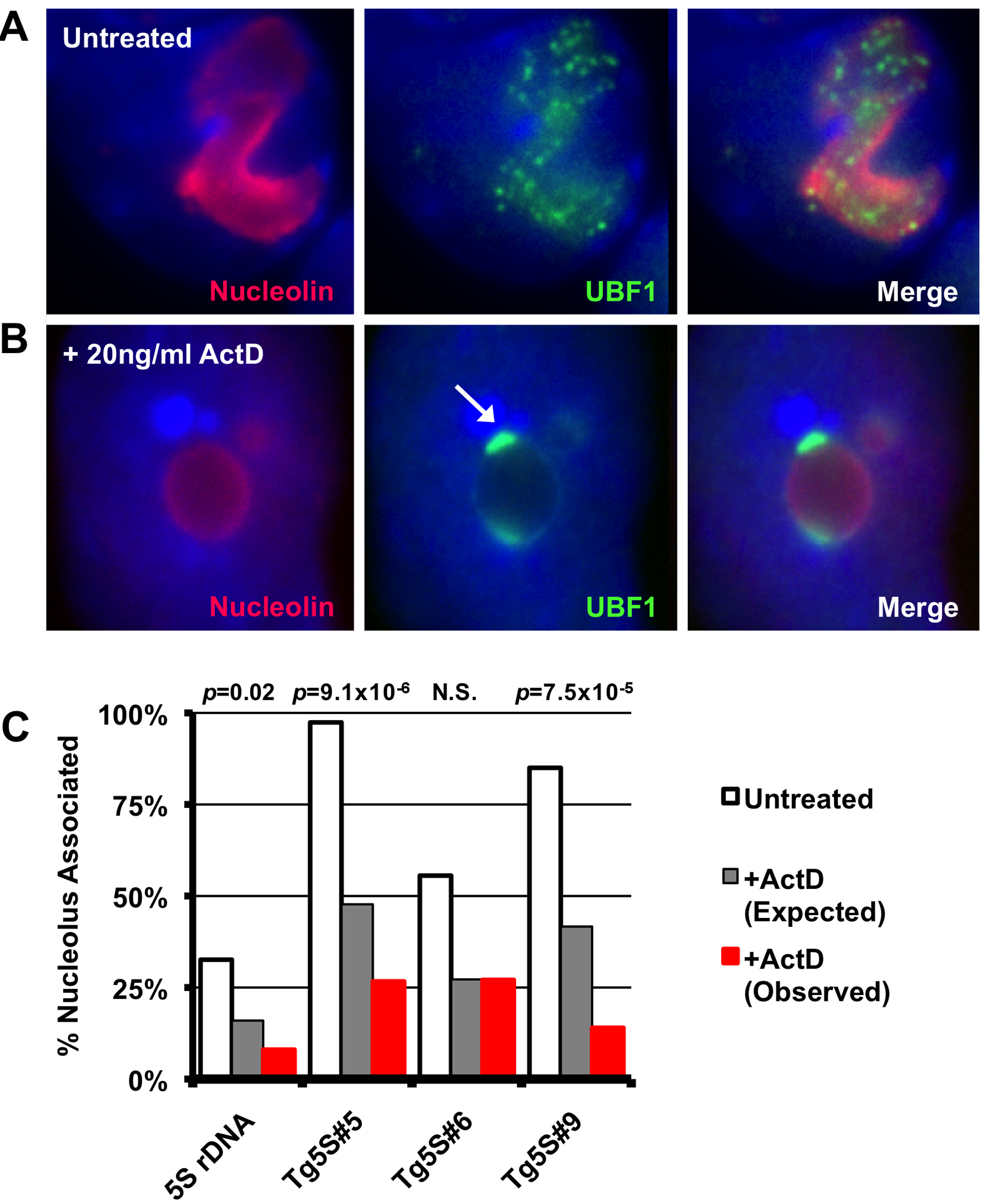

Supplement: Figure S2 — Pharmacological inhibition of RNA polymerase I (pol I) activity results in nucleolar reorganization and a decrease in 5S rDNA and Tg5S nucleolar association. ES lines were treated with a low dose of Actinomycin D (ActD; 20 ng/ml) for 2 hours prior to fixation to inhibit Pol I elongation. Note that ActD treatment results in redistribution of the pol I transcription factor UBF1(green) from intranucleolar foci (A) into focal concentrations at the nucleolar periphery (B, white arrow), and restructuring of nucleoli into a more spherical morphology. We measured the size of ActD-treated nucleoli to be 51% smaller than untreated nucleoli. C. Localization of 5S rDNA (n = 112) and Tg5S after ActD treatment (Tg5S#5, n = 30; Tg5S#6, n = 48; Tg5S#9, n = 50). To normalize for changes in nucleolar size, we calculated the ‘expected’ localization as the frequency of association in untreated cells by the relative nucleolar size in ActD treated cells. Statistical significance was determined by comparing the expected frequency to the observed frequency by chi-squared; N.S., not significant. (TIF) [file pgen.1002468.s002.tif]

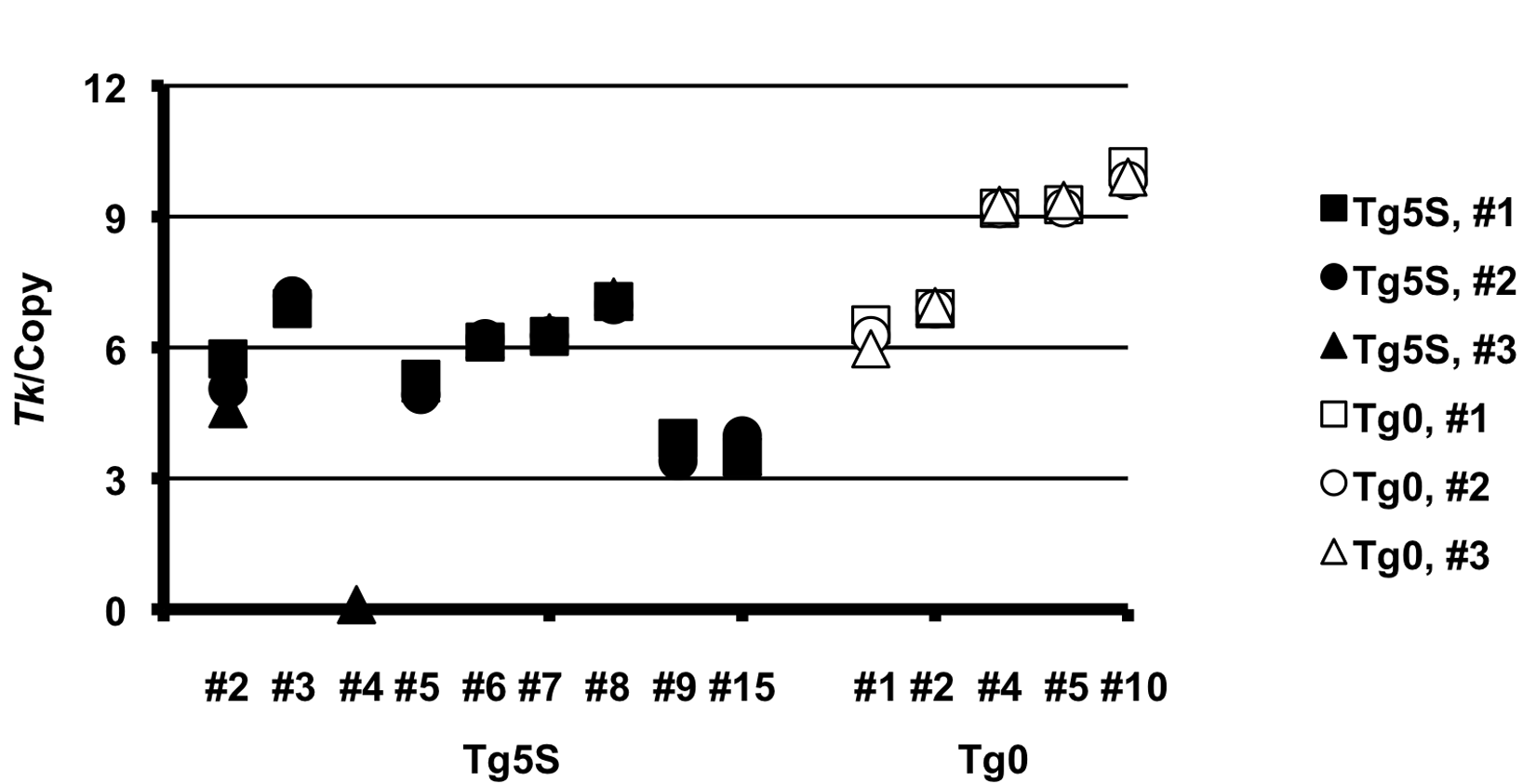

Supplement: Figure S3 — Normalized Tk shown for each individual line. Each technical replicate is shown as a different symbol. (TIF) [file pgen.1002468.s003.tif]

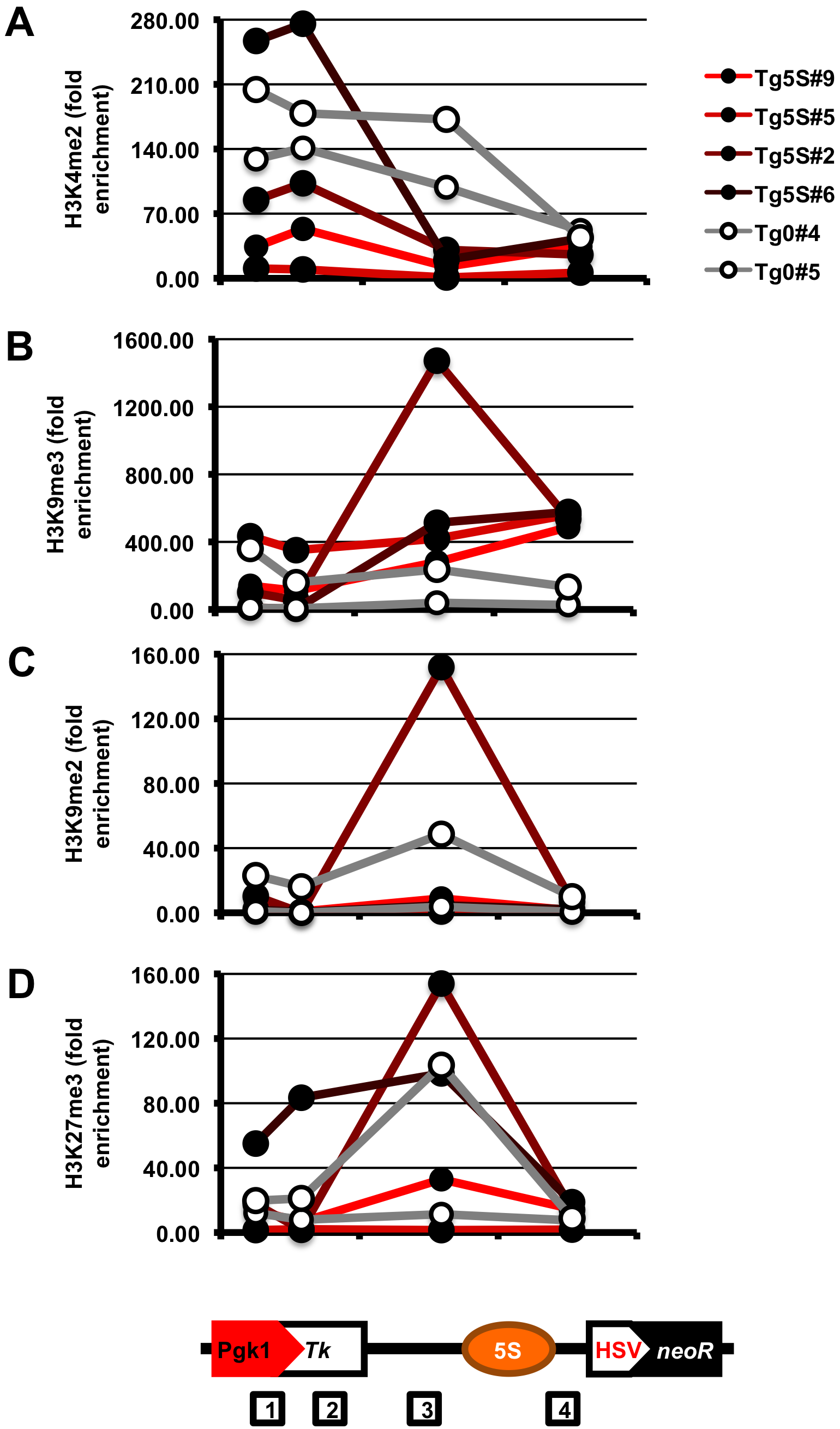

Supplement: Figure S4 — Quantification of histone modification enrichment over transgenes for (A) H3K4me2, (B) H3K9me3, (C) H3K9me2, and (D) H3K27me3. Values are represented as fold-enrichment relative with a negative control region lacking that modification. Also shown is a schematic of the transgene with positions of regions assayed. 2–3 replicates of each reaction were performed for each point. (TIF) [file pgen.1002468.s004.tif]

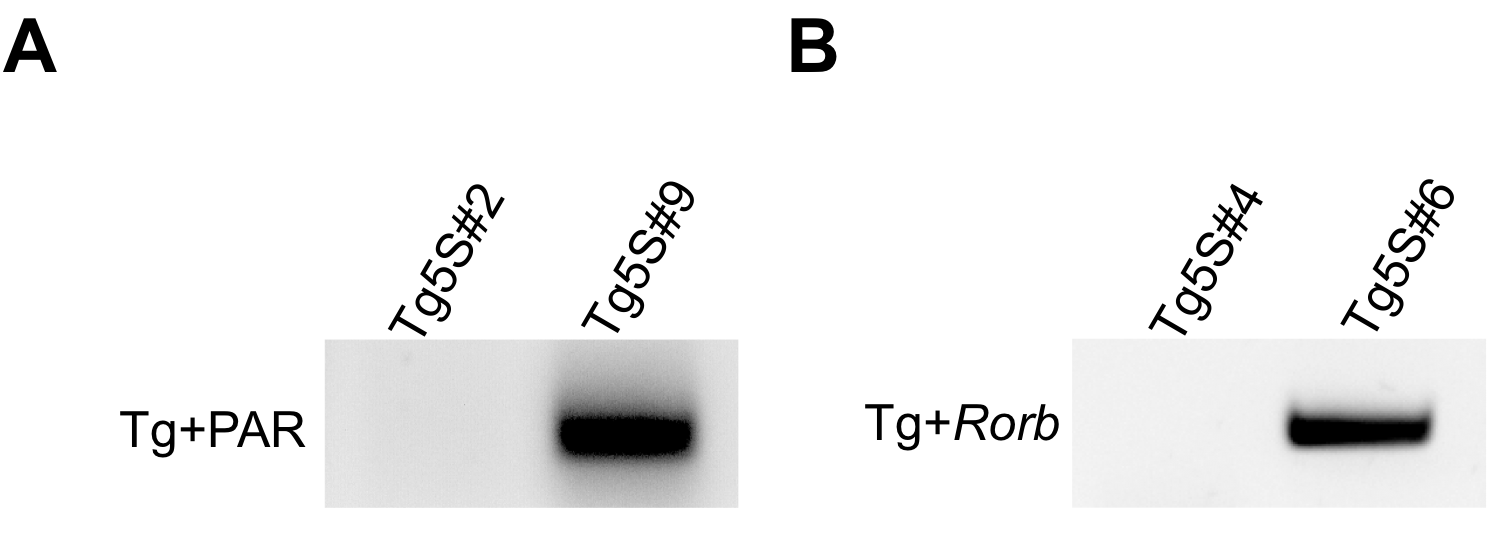

Supplement: Figure S5 — A. PCR assays to genotype PAR insertion in Tg5S#9; Tg5S#2 was used as a negative control. B. PCR assay to genotype insertion of the transgene into Rorb allele in Tg5S#6; Tg5S#4 is shown as a negative control. (TIF) [file pgen.1002468.s005.tif]

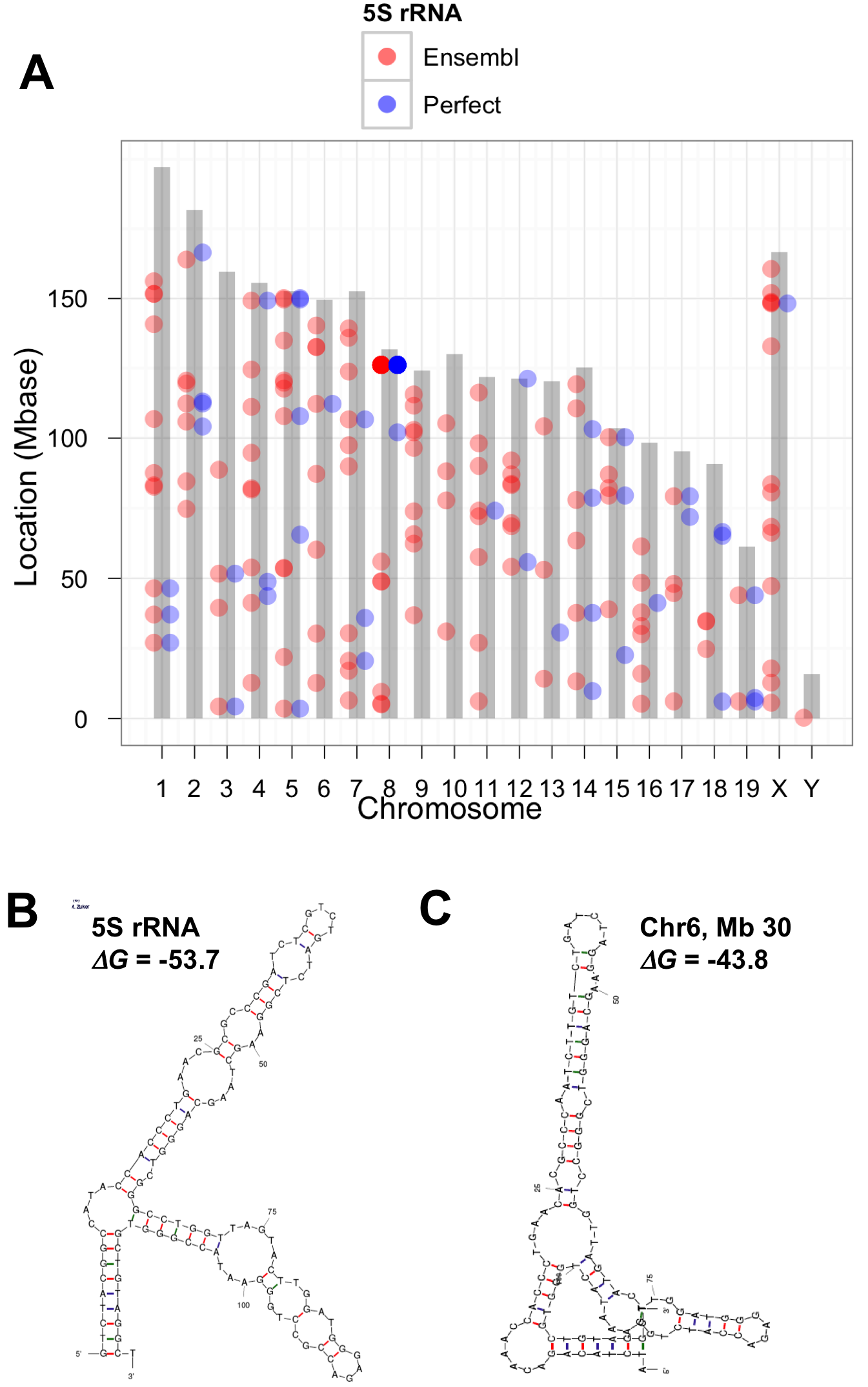

Supplement: Figure S6 — A. Distribution of 5S rDNA as annotated by in Ensembl (NCBIM37) (red dots). Perfect A and C boxes are shown as blue dots; note that a number of perfect A/C boxes are found outside of annotated 5S rDNA. The 5S rDNA array is located near the telomere of chromosome 8 (bold). Since the structure of 5S rRNA is highly conserved, we hypothesized that if the single genes were truly 5S rRNA, then they should form the expected structure. Using a folding algorithm (mfold, http://mfold.rna.albany.edu [36]), we predicted structure for all single 5S rDNA genes, and found that none had a structure resembling 5S rRNA, or thermodynamic stability (−ΔG), suggesting these elements are likely to be pseudogenes. Predicted 5S rRNA structure is shown in (B), while the structure of the most thermostable of 5S pseudogenes in (C). (TIF) [file pgen.1002468.s006.tif]

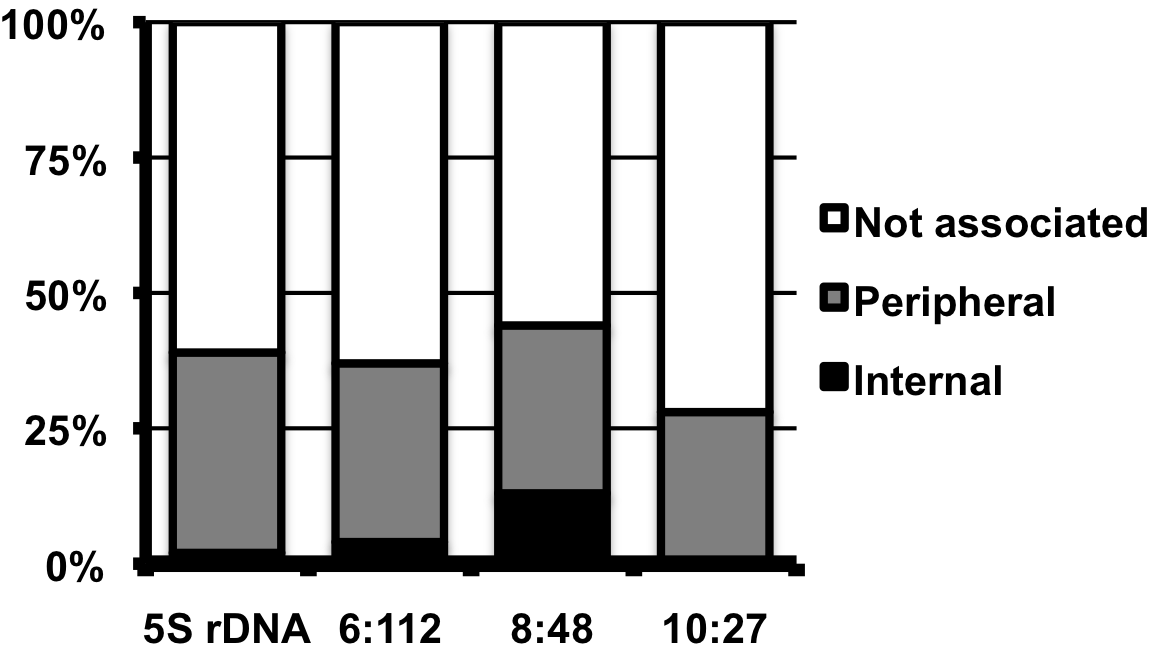

Supplement: Figure S7 — Nucleolar association of 5S rDNA (n = 83) and pseudogenes: 6∶112 (n = 49), 8∶48 (n = 61), and 10∶27 (n = 21). Pseudogenes are labeled by their location in the genome as chromosome∶megabase. For analysis, deconvolved Z-stacks were rendered as 3-dimensional models(see Methods). Frequency for localization of 8∶48 was comprable to analysis of single focal planes (see Figure 5B; 33% peripheral, 6% internal). (TIF) [file pgen.1002468.s007.tif]
