# Supplementary material for: Nucleolar Association and Transcriptional Inhibition through 5S rDNA in Mammals
Source: PLoS Genet. 2012 Jan 19;8(1):e1002468. doi: 10.1371/journal.pgen.1002468 (PMC3261910; doi:10.1371/journal.pgen.1002468)
Supplement: Table S1 — Mouse 5S Pseudogenes. Positional information of 5S rDNA psuedogenes in the mouse genome (based on the NCBI m37 mouse assembly). Also included are the percent identity of the A and C boxes, as well as the entire sequence, to the 5S rDNA consensus. (DOC) [file pgen.1002468.s008.doc]

Supplementary Table 1. Mouse 5S psuedogenes.

| **Chr:Mb** | **Ensembl ID** | **Chr** | **Start** | **Stop** | **Strand** | **A+C Identity** | **Overall Identity** | **Notes** |
| --- | --- | --- | --- | --- | --- | --- | --- | --- |
| 1:27 | ENSMUSG00000065125 | 1 | 27015948 | 27016066 | -1 | 100% | 90% |  |
| 1:37 | ENSMUSG00000070201 | 1 | 37062284 | 37062402 | 1 | 100% | 91% |  |
| 1:46 | ENSMUSG00000070199 | 1 | 46431042 | 46431160 | 1 | 100% | 93% |  |
| 1:60 | ENSMUSG00000065058 (NCBIM36) | 1 | 60459195 | 60459285 | 1 | 87% | 63% | no longer annotated; only as repeatmasker track. See NCBIM36 |
| 1:82 | ENSMUSG00000065055 | 1 | 82631588 | 82631685 | 1 | 96% | 87% |  |
| 1:87 | ENSMUSG00000064414 | 1 | 87571457 | 87571560 | 1 | 91% | 85% |  |
| 1:140 | ENSMUSG00000065275 | 1 | 140774839 | 140774954 | -1 | 91% | 67% |  |
| 1:151 | ENSMUSG00000077516 | 1 | 151688660 | 151688791 | 1 | 91% | 70% |  |
| 1:156 | ENSMUSG00000064860 | 1 | 156158538 | 156158657 | -1 | 96% | 72% |  |
| 2:76 | ENSMUSG00000077720 (NCBIM36) | 2 | 76353159 | 76353248 | -1 | 91% | 62% | no longer annotated; only as repeatmasker track. See NCBIM36 |
| 2:84 | ENSMUSG00000077382 | 2 | 84540905 | 84541017 | -1 | 87% | 61% |  |
| 2:112 | ENSMUSG00000077705 | 2 | 112309410 | 112309523 | -1 | 100% | 62% |  |
| 2:113 | ENSMUSG00000077580 (NCBIM36) | 2 | 113046101 | 113046198 | -1 | 100% | 80% | no longer annotated; only as repeatmasker track. See NCBIM36 |
| 2:119 | ENSMUSG00000064906 | 2 | 119456854 | 119456972 | 1 | 96% | 90% |  |
| 2:120 | ENSMUSG00000065083 | 2 | 120466447 | 120466571 | -1 | 96% | 74% |  |
| 2:141 | ENSMUSG00000065681 (NCBIM36) | 2 | 141458218 | 141458307 | 1 | 87% | 63% | no longer annotated; only as repeatmasker track. See NCBIM36 |
| 2:166 | ENSMUSG00000065871 (NCBIM36) | 2 | 166480556 | 166480644 | -1 | 100% | 71% | now annotated as miRNA:ENSMUSG00000088399 |
| 3:4 | ENSMUSG00000077912 | 3 | 4338998 | 4339116 | 1 | 100% | 95% |  |
| 3:39 | ENSMUSG00000064825 | 3 | 39534886 | 39535004 | -1 | 96% | 93% |  |
| 3:51 | ENSMUSG00000065070 | 3 | 51632834 | 51632950 | 1 | 100% | 80% |  |
| 3:88 | ENSMUSG00000065236 | 3 | 88710432 | 88710550 | 1 | 96% | 87% |  |
| 4:12 | ENSMUSG00000065823 | 4 | 12739853 | 12739959 | 1 | 91% | 89% |  |
| 4:41 | ENSMUSG00000065873 | 4 | 41250191 | 41250305 | 1 | 91% | 77% |  |
| 4:48 | ENSMUSG00000064383 (NCBIM36) | 4 | 48775748 | 48775833 | -1 | 100% | 71% | now annotated as miRNA:ENSMUSG00000088919 |
| 4:53 | ENSMUSG00000065887 | 4 | 53873524 | 53873642 | 1 | 96% | 95% |  |
| 4:149 | ENSMUSG00000064798 | 4 | 149191841 | 149191949 | 1 | 100% | 83% |  |
| 5:3 | ENSMUSG00000065044 | 5 | 3561154 | 3561273 | 1 | 100% | 88% |  |
| 5:21 | ENSMUSG00000065660 | 5 | 21936149 | 21936261 | -1 | 100% | 83% |  |
| 5:53 | ENSMUSG00000077316 | 5 | 53534399 | 53534477 | 1 | 91% | 60% |  |
| 5:53 | ENSMUSG00000077696 | 5 | 53737498 | 53737611 | -1 | 78% | 64% |  |
| 5:107 | ENSMUSG00000064554 | 5 | 107830338 | 107830456 | 1 | 100% | 85% |  |
| 5:120 | ENSMUSG00000064617 | 5 | 120514344 | 120514452 | 1 | 96% | 85% |  |
| 5:124 | ENSMUSG00000064946 (NCBIM36) | 5 | 124788418 | 124788527 | 1 | 87% | 65% | no longer annotated; only as repeatmasker track. See NCBIM36 |
| 5:134 | ENSMUSG00000077437 | 5 | 134892109 | 134892222 | 1 | 70% | 74% |  |
| 5:150 | ENSMUSG00000064704 | 5 | 150092694 | 150092799 | -1 | 100% | 83% |  |
| 6:12 | ENSMUSG00000065030 | 6 | 12692899 | 12693018 | 1 | 83% | 76% |  |
| 6:30 | ENSMUSG00000065662 | 6 | 30329101 | 30329214 | 1 | 65% | 89% |  |
| 6:60 | ENSMUSG00000064515 | 6 | 60200786 | 60200904 | 1 | 96% | 71% |  |
| 6:73 | ENSMUSG00000077556 (NCBIM36) | 6 | 73034933 | 73035024 | -1 | 91% | 72% | no longer annotated; only as repeatmasker track. See NCBIM36 |
| 6:112 | ENSMUSG00000077262 | 6 | 112184157 | 112184256 | -1 | 100% | 69% |  |
| 6:132 | ENSMUSG00000064832 | 6 | 132514990 | 132515110 | 1 | 100% | 62% |  |
| 6:132 | ENSMUSG00000065161 | 6 | 132541076 | 132541196 | 1 | 100% | 63% |  |
| 6:140 | ENSMUSG00000065882 | 6 | 140268151 | 140268254 | 1 | 96% | 89% |  |
| 7:17 | ENSMUSG00000065893 | 7 | 17057162 | 17057280 | 1 | 96% | 95% |  |
| 7:20 | ENSMUSG00000070150 | 7 | 20595433 | 20595551 | -1 | 100% | 83% |  |
| 7:30 | ENSMUSG00000065385 | 7 | 30442180 | 30442295 | -1 | 70% | 81% |  |
| 7:106 | ENSMUSG00000065311 | 7 | 106646228 | 106646332 | 1 | 100% | 81% |  |
| 7:135 | ENSMUSG00000077283 | 7 | 135918360 | 135918477 | -1 | 39% | 53% |  |
| 8:5 | ENSMUSG00000064962 | 8 | 5069259 | 5069377 | -1 | 96% | 89% |  |
| 8:5 | ENSMUSG00000064507 | 8 | 5332995 | 5333113 | -1 | 96% | 76% |  |
| 8:9 | ENSMUSG00000064618 | 8 | 9568132 | 9568249 | -1 | 96% | 77% |  |
| 8:48 | ENSMUSG00000064508 | 8 | 48804600 | 48804723 | 1 | 100% | 70% |  |
| 8:48 | ENSMUSG00000064519 | 8 | 48984536 | 48984648 | 1 | 91% | 89% |  |
| 8:88 | ENSMUSG00000077721 (NCBIM36) | 8 | 88720785 | 88720864 | -1 | 91% | 70% | no longer annotated; only as repeatmasker track. See NCBIM36 |
| 9:36 | ENSMUSG00000077401 | 9 | 36833984 | 36834092 | 1 | 87% | 81% |  |
| 9:53 | ENSMUSG00000064980 (NCBIM36) | 9 | 53507910 | 53508034 | 1 | 87% | 87% | no longer annotated; only as repeatmasker track. See NCBIM36 |
| 9:65 | ENSMUSG00000065387 | 9 | 65684160 | 65684272 | 1 | 83% | 80% |  |
| 9:73 | ENSMUSG00000064744 | 9 | 73907146 | 73907264 | 1 | 91% | 86% |  |
| 9:102 | ENSMUSG00000065107 | 9 | 102030218 | 102030335 | 1 | 100% | 90% |  |
| 9:102 | ENSMUSG00000065466 | 9 | 102913747 | 102913850 | -1 | 78% | 86% |  |
| 9:111 | ENSMUSG00000077156 | 9 | 111516402 | 111516519 | -1 | 96% | 65% |  |
| 9:113 | ENSMUSG00000065120 (NCBIM36) | 9 | 113984521 | 113984587 | 1 | 96% | 75% | no longer annotated; only as repeatmasker track. See NCBIM36 |
| 9:115 | ENSMUSG00000065149 | 9 | 115535718 | 115535836 | -1 | 91% | 85% |  |
| 10:27 | ENSMUSG00000065099 (NCBIM36) | 10 | 27671642 | 27672666 | 1 | 83% | 70% | now annotated as miRNA:ENSMUSG00000087824 |
| 10:77 | ENSMUSG00000065691 | 10 | 77776910 | 77777028 | 1 | 91% | 86% |  |
| 10:77 | ENSMUSG00000089533 | 10 | 77981368 | 77981483 | 1 | 87% | 78% |  |
| 10:88 | ENSMUSG00000065005 | 10 | 88172753 | 88172872 | -1 | 70% | 74% |  |
| 11:6 | ENSMUSG00000077524 | 11 | 6173314 | 6173427 | 1 | 91% | 79% |  |
| 11:57 | ENSMUSG00000065806 | 11 | 57577929 | 57578047 | 1 | 83% | 79% |  |
| 11:74 | ENSMUSG00000070178 | 11 | 74133294 | 74133412 | 1 | 100% | 94% |  |
| 11:90 | ENSMUSG00000077706 | 11 | 90046323 | 90046441 | -1 | 83% | 57% |  |
| 11:116 | ENSMUSG00000064588 | 11 | 116187916 | 116188031 | -1 | 87% | 70% |  |
| 12:54 | ENSMUSG00000070192 | 12 | 54147695 | 54147813 | -1 | 96% | 90% |  |
| 12:55 | ENSMUSG00000065133 (NCBIM36) | 12 | 55767687 | 55767777 | -1 | 100% | 80% | now annotated as miRNA:ENSMUSG00000088962 |
| 12:68 | ENSMUSG00000065233 | 12 | 68590100 | 68590218 | -1 | 91% | 87% |  |
| 12:83 | ENSMUSG00000064714 | 12 | 83251455 | 83251550 | 1 | 96% | 81% |  |
| 12:87 | ENSMUSG00000075973 | 12 | 87059427 | 87059545 | -1 | 96% | 93% |  |
| 12:92 | ENSMUSG00000064548 | 12 | 92040059 | 92040175 | 1 | 96% | 87% |  |
| 12:121 | ENSMUSG00000065671 (NCBIM36) | 12 | 121157828 | 121157914 | -1 | 100% | 77% | now annotated as miRNA:ENSMUSG00000088807 |
| 13:14 | ENSMUSG00000077802 | 13 | 14211010 | 14211122 | -1 | 83% | 78% |  |
| 13:53 | ENSMUSG00000065771 | 13 | 53059990 | 53060101 | -1 | 83% | 64% |  |
| 13:58 | ENSMUSG00000064635 (NCBIM36) | 13 | 58541206 | 58541303 | -1 | 96% | 85% | no longer annotated; only as repeatmasker track. See NCBIM36 |
| 13:104 | ENSMUSG00000064455 | 13 | 104186282 | 104186386 | 1 | 96% | 81% |  |
| 14:37 | ENSMUSG00000065894 | 14 | 37669115 | 37669230 | -1 | 100% | 72% |  |
| 14:110 | ENSMUSG00000065102 | 14 | 110561057 | 110561167 | 1 | 96% | 73% |  |
| 14:119 | ENSMUSG00000077661 | 14 | 119226131 | 119226253 | -1 | 91% | 61% |  |
| 15:79 | ENSMUSG00000064409 | 15 | 79522932 | 79523055 | 1 | 100% | 64% |  |
| 15:82 | ENSMUSG00000064959 | 15 | 82173083 | 82173201 | 1 | 96% | 87% |  |
| 15:86 | ENSMUSG00000064631 | 15 | 86999543 | 86999646 | 1 | 78% | 76% |  |
| 16:16 | ENSMUSG00000077350 | 16 | 16010993 | 16011102 | -1 | 78% | 55% |  |
| 16:33 | ENSMUSG00000070225 | 16 | 33001989 | 33002107 | 1 | 96% | 96% |  |
| 16:41 | ENSMUSG00000064903 (NCBIM36) | 16 | 41217853 | 41217944 | 1 | 100% | 77% | now annotated as miRNA:ENSMUSG00000088109 |
| 16:48 | ENSMUSG00000064932 | 16 | 48410419 | 48410537 | -1 | 87% | 75% |  |
| 16:61 | ENSMUSG00000064467 | 16 | 61372853 | 61372970 | -1 | 83% | 78% |  |
| 17:6 | ENSMUSG00000077789 | 17 | 6147419 | 6147540 | 1 | 87% | 60% |  |
| 17:44 | ENSMUSG00000070209 | 17 | 44777974 | 44778092 | -1 | 78% | 73% |  |
| 17:47 | ENSMUSG00000064748 | 17 | 47985783 | 47985901 | 1 | 91% | 94% |  |
| 17:79 | ENSMUSG00000064770 | 17 | 79258274 | 79258392 | 1 | 100% | 77% |  |
| 16:6 | ENSMUSG00000064474 (NCBIM36) | 18 | 6085369 | 6085455 | 1 | 100% | 72% | now annotated as miRNA:ENSMUSG00000089200 |
| 18:34 | ENSMUSG00000064857 | 18 | 34807054 | 34807172 | 1 | 96% | 93% |  |
| 19:35 | ENSMUSG00000077313 (NCBIM36) | 19 | 35680207 | 35680293 | 1 | 83% | 61% | now annotated as miRNA:ENSMUSG00000089494 |
| X:5 | ENSMUSG00000075918 | X | 5684009 | 5684127 | 1 | 96% | 96% |  |
| X:12 | ENSMUSG00000065813 | X | 12792824 | 12792935 | -1 | 100% | 79% |  |
| X:17 | ENSMUSG00000065068 | X | 17900570 | 17900688 | -1 | 96% | 90% |  |
| X:66 | ENSMUSG00000065689 | X | 66202739 | 66202859 | -1 | 100% | 88% |  |
| X:68 | ENSMUSG00000077309 | X | 68384412 | 68384529 | 1 | 91% | 57% |  |
| X:80 | ENSMUSG00000077620 | X | 80591082 | 80591198 | 1 | 83% | 66% |  |
| X:83 | ENSMUSG00000065758 | X | 83651467 | 83651585 | -1 | 91% | 86% |  |
| X:106 | ENSMUSG00000064521 (NCBIM36) | X | 106295123 | 106295213 | -1 | 96% | 73% | no longer annotated; only as repeatmasker track. See NCBIM36 |
| X:132 | ENSMUSG00000065719 | X | 132812044 | 132812162 | 1 | 78% | 81% |  |
| X:148 | ENSMUSG00000070219 | X | 148212214 | 148212332 | 1 | 100% | 89% |  |
| X:148 | ENSMUSG00000084637 | X | 148419853 | 148419959 | 1 | 65% | 75% |  |
| X:148 | ENSMUSG00000084584 | X | 148738544 | 148738654 | 1 | 83% | 77% |  |
| X:151 | ENSMUSG00000077743 | X | 151977833 | 151977951 | 1 | 87% | 68% |  |
| X:155 | ENSMUSG00000065867 (NCBIM36) | X | 155887204 | 155887302 | 1 | 96% | 72% | now annotated as miRNA:ENSMUST00000157212 |
| Y:0 | ENSMUSG00000075874 | Y | 328073 | 328191 | -1 | 100% | 94% |  |
