# Supplementary material for: Nucleolar Association and Transcriptional Inhibition through 5S rDNA in Mammals
Source: PLoS Genet. 2012 Jan 19;8(1):e1002468. doi: 10.1371/journal.pgen.1002468 (PMC3261910; doi:10.1371/journal.pgen.1002468)
Supplement: Table S2 — PCR primers used in this study. (DOC) [file pgen.1002468.s009.doc]

Supplementary Table 2. PCR primers used in this study.

| **Assay** | **Fwd/ Rev** | **Sequence (5’-3’)** | **Notes** |
| --- | --- | --- | --- |
| *Neo* | Fwd | AGACAATCGGCTGCTCTGAT | RT PCR and Copy Number |
|  | Rev | ATACTTTCTCGGCAGGAGCA |  |
| *Tk* | Fwd | GGCCATGGTCACCTAGAGAA | RT PCR |
|  | Rev | TTGAGGCCCTTGAGTAATGG |  |
| *Gapdh* | Fwd | GGAAGCAGCATTCAGGTCTCT | RT PCR |
|  | Rev | CCTCCTCCCTCTCTTTGGAC |  |
| Inverse PCR | Fwd | AATTCGCCAATGACAAGACG | 1^st^ round |
|  | Rev | CGTTGGCTACCCGTGATATT |  |
| Inverse PCR | Fwd | CTGGGAATACCAGGTGTCGT | 2^nd^ round |
|  | Rev | TCGCCTTCTTGACGAGTTCT |  |
| TAIL PCR:1st Round | Rev | GCATGCCCGACGGCGAGGATCT | Gene Specific primers; used Fwd primers as described in Pillai *et al.,* 2008 |
| TAIL PCR:2nd Round | Rev | GACTGTGGCCGGCTGGGTGTGG |  |
| TAIL PCR:3rd Round | Rev | TTGGCGGCGAATGGGCTGACCG |  |
| Genotyping of Tg5S#9 | Fwd | CTCTCCAGACCCCTTCATCA |  |
|  | Rev | TCGTTTGTTCGGATCATTCA |  |
| Genotyping of Tg5S#6 | Fwd | ATCGCCTTCTATCGCCTTCT |  |
|  | Rev | CTGCATCCCACAGTCACACA |  |
| Tg-5S rDNA | Fwd | CTGGGAATACCAGGTGTCGT | ChIP of transgene-5S rDNA |
|  | Rev | GAGTCAGTGAGCGAGGAAGC |  |
| *Ascl2* promoter | Fwd | ATTTAGCCCCTCATGCACAC | Normalization for ChIP and Copy Number |
|  |  | TCCAGGAACCGAACAAAAAC |  |
|  |  |  |  |
| **5S/rDNA pseudogene ChIP primers** | | |  |
| 5SF | Fwd | GTC TAC GGA CAT ACC ACC CTG AAC G |  |
| 5SR | Rev | TAC AGC ACC CGG TAT TCC CAG GCG |  |
| 2:76 | Fwd | ATC TAT GAC TAT GCC ACC CTG G |  |
| 2:76 | Rev | GT AAG AAG TTC TGA AAT CTC |  |
| 5:134 | Fwd | AGC CAT CCC ACC CCA GTT TC |  |
| 5:134 | Rev | CCT ATA GCA CCT AGC ACC TAC |  |
| 6:30 | Fwd | CAT CTC ATC TAC AGA CAA ACC |  |
| 6:30 | Rev | TT AAA GCC TAG AGC ACC CAG |  |
| 6:112 | Rev | CAT CCT CTT CCA TCA GCT CTC | use 5SF as forward primer |
| 7:30 | Fwd | GTC TAT GGC GGT AAC TAC CCT C |  |
| 7:30 | Rev | TAA AAG CCT ACT ACA CCC AG |  |
| 8:48 | Fwd | CTC TAC GGC CAT ACC ACT CT | ENSMUSG00000064508 |
| 8:48 | Rev | AAG TGG CCA GAG CTT AAG CTG |  |
| 10:27 | Fwd | GAAT ATC GAT GCA GTC TAT GG |  |
| 10:27 | Rev | TGA GTA TTT TTG CAT CAA CAT |  |
| 11:116 | Fwd | TCT GCT GAG GCT TCC ATC CTG |  |
| 11:116 | Rev | CTA AGC CTA TTC TGC TCA CTA |  |
| 11:74 | Rev | TAC AGC ACC CGG TAT TCC CAG GCA | use 5SF as forward primer |
| 14:110 | Fwd | GTC TAC CGC CAT ACC ACC CA |  |
| 14:110 | Rev | CCC TTC AGG TGC CAG TCC TT |  |
| 17:79 | Fwd | CC TAC AGC ACC TGG TAT TCC CAG GT |  |
| 17:79 | Rev | AAC TAC CAA GTG AAG AAT GAA AAC G |  |
| X:12 | Fwd | TTC TCT  GGC CAT ACC ACC CTG AAC G |  |
| X:12 | Rev | GTG TGG TGG CAC ATG CAG ATT TCT C |  |
| X:66 | Fwd | GTCTATGGCCATACCACCCTG |  |
| X:66 | Rev | GCCTACAACTCCAGGTATTCG |  |
| Y:0 | Fwd | GTC TAC GGC CAT AAC ACC CT |  |
| Y:0 | Rev | TAC AGC ACC CTG TAT TCC CAG GCA |  |
|  |  |  |  |
| **Tg5S/Tg0 ChIP primers** | |  |  |
| 1 | Fwd | CCGTACGTCGGTTGTTATGG | *Tk* promoter |
| 1 | Rev | CGCTGTTCTCCTCTTCCTCA |  |
| 2 | Fwd | ACACCCGCCAGTAAGTCATC | *Tk* gene body |
| 2 | Rev | CAGAAAATGCCCACGCTACT |  |
| 3 | Fwd | AATTCGCCAATGACAAGACG | Vector backbone |
| 3 | Rev | CGAAGTTATGAATTTCGAGCAG |  |
| 4 | Fwd | CCACAGTCGATGAATCCAGA | distal to 5S cloning site |
| 4 | Rev | AGCACGTACTCGGATGGAAG |  |
| *Oct4* | Fwd | ATCCGAGCAACTGGTTTGTG | *Pou5f1* promoter; negative control for H3K9me2, H3K9me3, and H3K27me3 ChIP |
|  | Rev | AGCGCTATCTGCCTGTGTCT |  |
| Non-genic | Fwd | TTTCCTGCCTCTGCCTTTTA | Negative contol for H3K4me2 ChIP; chromosome 6 |
|  | Rev | AGGTGGCACAGTGGGTAAAG |  |
